# Cardiovascular stents: overview, evolution, and next generation

**DOI:** 10.1007/s40204-018-0097-y

**Published:** 2018-09-10

**Authors:** Setareh Borhani, Shadi Hassanajili, Seyed Hossein Ahmadi Tafti, Shahram Rabbani

**Affiliations:** 1 Department of Chemical Engineering, School of Chemical and Petroleum Engineering, Shiraz University, Shiraz, Iran; 2 Department of Nanochemical Engineering, School of New Science and Technology, Shiraz University, Shiraz, Iran; 3 Research Center for Advanced Technologies in Cardiovascular Medicine, Tehran Heart Center, Tehran University of Medical Sciences, North Kargar, Tehran, Iran

**Keywords:** Coronary artery diseases, Cardiovascular stents, In-stent restenosis, Stent thrombosis

## Abstract

Compared to bare-metal stents (BMSs), drug-eluting stents (DESs) have been regarded as a revolutionary change in coronary artery diseases (CADs). Releasing pharmaceutical agents from the stent surface was a promising progress in the realm of cardiovascular stents. Despite supreme advantages over BMSs, in-stent restenosis (ISR) and long-term safety of DESs are still deemed ongoing concerns over clinically application of DESs. The failure of DESs for long-term clinical use is associated with following factors including permanent polymeric coating materials, metallic stent platforms, non-optimal drug releasing condition, and factors that have recently been supposed as contributory factors such as degradation products of polymers, metal ions due to erosion and degradation of metals and their alloys utilizing in some stents as metal frameworks. Discovering the direct relation between stent materials and associating adverse effects is a complicated process, and yet it has not been resolved. For clinical success it is of significant importance to optimize DES design and explore novel strategies to overcome all problems including inflammatory response, delay endothelialization, and sub-acute stent thrombosis (ST) simultaneously. In this work, scientific reports are reviewed particularly focusing on recent advancements in DES design which covers both potential improvements of existing and recently novel prototype stent fabrications. Covering a wide range of information from the BMSs to recent advancement, this study mostly sheds light on DES’s concepts, namely stent composition, drug release mechanism, and coating techniques. This review further reports different forms of DES including fully biodegradable DESs, shape-memory ones, and polymer-free DESs.

## Introduction

Heart disease involves one of the problems with valves, muscles or coronary arteries all of which cause the heart not to function well. Blood carries nutrients and oxygen with itself and the reduced ability of the heart to circulate enough amount of blood within vessels and arteries leads to a crisis in the body. CAD is a common heart disease among patients with heart disease, and it arises from the buildup of plaque on the inner surface of arteries. The blockage of the artery by plaque accumulation is called atherosclerosis. In a developed case the artery becomes significantly narrowed and in a worst case it is blocked (Driver 2012). The first use of stent for clinical practice was introduced to reduce the risk of percutaneous transluminal coronary angioplasty (PTCA). PTCA was utilized in narrowed arteries to re-open them. It makes use of a catheter with an installed folded balloon which is inflated after the deliverability in the narrowed part of the artery. The inflated balloon compresses the obstructing plaque which causes the obstructed inner wall to be enlarged. Under high stress the device intensifies the risk of thrombosis due to the injury made by catheter implantation and balloon expansion. The idea of cardiovascular stent, afterward, has revolutionized the treatment of coronary-related diseases (Driver 2012; Guildford et al. 2010). Stents are the wise replacement for surgery that was first introduced with balloon angioplasty (BA) in 1977 by Grüntzig (1978). This trend continued by the first human implantation of a self-expanding stent in 1986 (Sigwart et al. 1987) and further in 1987 (Palmaz et al. 1987), by the first human implantation of a balloon-expandable stent (Grabow et al. 2010).

Generally, stents are tubular implants to give the stenotic arteries or other non-vascular conduits a mechanical strength until the risk of fully closure be removed. There are two groups of stents, as stated earlier, self-expanding and balloon-expandable stents. Early stents were mostly fabricated from metals (until very recently); hence the first-generation of stents are called bare-metal stents. These permanent metallic frameworks are made of stainless steel and cobalt-chromium (CoCr) alloys for balloon-expandable, and nickel-titanium alloys (nitinol) for self-expanding stents (Grabow et al. 2010; Hanawa 2009). Although the revolution was considered as a turning point in the field of surgery, it had its own disadvantages of increased risk of thrombosis and restenosis. During stenting procedures, it is very likely for intravascular injuries to happen which lead to in-stent restenosis (ISR). ISR is the leading cause of artery blockage over time, and the loss of artery patency subsequently causes stent failure. Cascade events lead to the loss of the artery patency which are as follows: Dysfunctional vascular endothelium directly causes ISR, and this starts to happen when there is the lack of antithrombic and antiatherogenic properties. The artery dysfunction in suppressing vascular smooth muscle cells’ (VSMCs) proliferation makes VSMCs overgrow inward the blood vessel. This results in the blockage of the vessel overtime (Nuhn et al. 2017; Guildford et al. 2010). As early reports suggested (Fischman et al. 1994), in about 15–20% of all implanted stents patients required re-intervention within 6–12 months after the first BMS implantation due to ISR. Generally, all kinds of stents have still been dealing with this problem. The rate of in-stent restenosis has been reduced after the arrival of new generations of stent family through recent advancement in this technology. However, the problem is still active. This review discusses different techniques for the stent design.

## Drug-eluting stents

### Permanent polymer-coated drug-eluting stents

The formation of blood clot in the stent has been classified into two groups: (1) acute stent thrombosis (ST) due to inadequate stent expansion or arterial dissection occurs mostly within hours of the stent implantation, (2) sub-acute late ST which occurs up to 30 days after the implantation (Gandhi and Dawkins 1999). The first week is said to be the highest risk period for implantation (Liu et al. 2014). To put the cascade biological events in order, the following 3-phase biological response is seen: (1) the inflammatory reaction occurs within 3–7 days, and the intimal thickness increases significantly in 4 weeks, (2) in the second phase, within 1–3 months, the stent surface is exposed to the blood flow within the vessel and the surrounding tissue starts to cover the stent surface. Despite the healing process in the second phase, there is still the risk of delayed or incomplete endothelialization which is the cause of thrombosis and restenosis, and (3) in the third phase, the stent is fully covered by the vascular tissue after three months of implantation process, but the risk of late ST is still alive due to the adverse material–tissue interactions. The risk of adverse biological responses in each phase needs to be prevented by a time-ordered drug release to the site of injury from a stent known as DES (Liu et al. 2014). With respect to drug-eluting stents, a suitable pharmaceutical agent is non-toxic, stable, compatible with the coating layer, and has appropriate mode of action (Driver 2012). For metallic DESs there is a limitation in delivering sufficient amount of drugs in a suitable time frame. They can only elute small amounts of drug without the ability of dual- or triple-eluting from the surface over a short period of time frame that is mostly not sufficient (Kohn and Zeltinger^2005^). For better loading drug molecules on the stent surface and for enhancing an engineered control over drug release, polymeric coatings have been developed as the new replacing technology. Polymer layers on the stent surface have the following roles: (1) inhibiting the drug from being washed off from the stent surface, (2) providing a suitable scaffold for drug loading, (3) providing an engineered control over the drug release, and (4) making the polymer coating a satisfactory platform with respect to biocompatibility after the drug has washed out (Driver 2012). It is vitally important to have deep insights into the drug release behavior in the physiological medium as an efficient healing technique. In addition, a time-programmed drug release should make a balance between the drug release to the artery and the drug absorption by the surrounding artery tissue. The rapid rate of drug release might exceed the tissue uptake. The slow release rate, on the other hand, could delay in tissue healing process (Balakrishnan et al. 2007). An ideal DES has a slow and controlled drug elution with a programmed release regarding the 3-phase adjusted remedy (Hu et al. 2015). Drugs for DES must have selective mode of action including: (1) capable of inhibiting the platelet aggregation, inflammation, smooth muscle cell (SMC) proliferation and migration, all of which finally tend to in-stent restenosis, (2) promoting appropriate healing and fast endothelialization (Ferns and Avades 2000).

First-generation DESs consisted of three main parts: a permanent metallic platform (mainly stainless steel), a permanent polymeric coating on the platform (that was loaded with anti-proliferative and/or anti-inflammatory therapeutic agents), and an active pharmaceutical agent which was incorporated into the polymeric coating (that was eluted from the polymeric layer) (Simard et al. 2014). Using early DESs was an efficient method to decrease the rate of re-intervention, which was due to the release of either anti-proliferative or immunosuppressant drugs on the site of vascular injury (Kraitzer et al. 2008). This technique has outperformed BMSs in reducing neointimal proliferation and restenosis based on clinical studies (Chen et al. 2015). Despite all previous facts, first-generation DESs are deviating from today’s medical standards (Simard et al. 2014). Serious clinical events cast doubt on the efficiency of DES in terms of long-term safety with increased risks of late and very late stent thrombosis. They have investigated some technical problems with DES which are as follows. First, delayed endothelialization caused by the locally delivered drugs. Second, inherent thrombogenicity of the stent as a foreign device to the immune system. Third, hypersensitivity and inflammatory reactions as a result, either due to the metal-based framework and/or polymeric coatings decorated on the stent surface to carry therapeutic agents. Fourth, insufficient drug amount in addition to lack of sustained drug release. Fifth, stent displacement (Saleh et al. 2017). DESs utilized anti-restenotic drugs with improved long-term safety as further advancement (Chen et al. 2015). They utilized a metal stent which was covered with a polymer film. First-generation DESs composed of a CoCr alloy platform. This substituted platform exhibited higher radial strength and improved radio-opacity in comparison to 316L stainless steel. Superior mechanical strength resulted in thinner stent struts which decreased the rate of restenosis after implantation (Chen et al. 2015). The first commercial DES, Cypher, was launched by Cordis (a Johnson & Johnson Company) in 2002. This drug-eluting stent was devised for the controlled release of sirolimus (anti-proliferative and immunosupressive) or paclitaxel (PTX) (anti-proliferative) from a non-degradable polymer coating layer. The polymer coating layers included poly(ethylene-*co*-vinyl acetate) (PEVA) and poly(*n*-butyl methacrylate) (PBMA) which were used as a platform for drug release. The stents were comprised of three separate layers of polymer: first, a parylene layer which was applied to the metal surface of the stent to ease the attachment of further layers by enhancing the adhesion to the surface. Second, the mixture of the drug with PEVA and PBMA (these two polymers are miscible with the drug) to allow controlled drug release. The outermost layer of PEVA/PBMA contained no drug, but was only applied to help drugs to have a controlled and sustained elution from the second layer. The PEVA/PMBA was designed for the best distributing pharmaceutical agents through the coating layer. Moreover, the mixing polymer layer provided a reservoir in which drug (sirolimus) diffuses through into the site of injury. The top coating layer was employed for inhibiting any burst release of drugs in order to have a longer drug elution to the site of action. It is important to deliver sufficient amount of drug in a desired time frame (Driver 2012). A stent undergoes mechanical constraints after implantation which results in a stent coating defect such as cracking, flaking, and delamination. Findings demonstrated requirements for a drug-eluting stent as follows (Farah 2018): First, the flexibility of a DES to make it possible to stretch without delamination or falling the stent apart (Tugtekin et al. 2004). Second, the polymer is responsible to place drug agents in its structure so that therapeutic agents could be released at a sustained, controlled, and predictable rate (Levy et al. 2009a). Third, the key properties of a polymer applied as an implant in the human body must include adjusted physical properties, stability, biocompatibility with vascular tissue, chemical compatibility with drugs, and the capability to control drug release (Parker et al. 2010). It is difficult to achieve a polymer with all desired properties, thus taking advantage of mixtures of polymers could help. However, due to unique properties of each polymer in the mixture working with this new chemical composition has its own limitations (Farah 2018). Although the coating polymer layer utilized in DESs empowered these stents with sustainable drug release, the long-term persistency of non-degradable polymers in the site of injury triggered adverse effects which led to late ST (Simard et al. 2014). The methodologies on which manufacturing of DES are conducted are mostly based on mechanical techniques such as dip coating and spray coating. This type of coating technique generates coatings with poor stability, fast drug release from the stent surface, and uncertainty about long-term safety (Farah 2018; Levy et al. 2009b). Biodegradable polymers were suggested as a new coating agent in order to avoid adverse pathological effects in addition to better controlled drug elution (Chen et al. 2015). This biodegradable polymer-coated DES has made a revolutionary transformation in the second generation of DES.

### Biodegradable polymer-coated drug-eluting stents

The advent of DES technology has brought its advantages to overcome the conventional limitations of BMS. Despite DES’s dramatic influence on clinical practice in vascular intervention, the incomplete endothelialization and hypersensitivity reactions to the polymer coating were major subjects of debate over the risk of implantation (Grabow et al. 2010). All drawbacks caused the first-generation DES to fail. Late thrombosis and delayed healing were two potential risks associating with the use of DES. Furthermore, their long-term efficiency was questionable since their coating material was not biodegradable and reports of hypersensitivity to DES implantation were reported (Farb et al. 2003; Joner et al. 2006; Lanzer et al. 2008; Nebeker et al. 2006; Virmani et al. 2004a, b). The long-term presence of non-biodegradable materials in stents leads to late complications such as thrombosis, neointimial hyperplasia, and chronic inflammation (Jiang et al. 2017). It was evident that the newly improved version should have the polymer coating with higher biocompatibility and probable degradability with improved pharmacologic action (Grabow et al. 2010). To circumvent the problems associated with durable polymer-coated DES, the modified second-generation DES made use of biodegradable polymer to enhance the clinical performance. This new treatment has the following properties: (1) the ability to deliver greater amounts of drug over a longer period of time, (2) the ability to prepare a suitable framework for loading various drugs if necessary, and (3) the ability to overcome unfavorable effects of stenting (Kohn and Zeltinger 2005; Tanguay et al. 1994). Van der Giessen et al. (1992) published their pioneering experimental studies of implanting non-biodegradable polyethylene-terephthalate braided mesh stent in animal models. The first trial for creating a biodegradable scaffold started later in 1996 when the efficiency of five different biodegradable polymers consisting poly(glycolic acid)/poly(lactic acid) PGA/PLA copolymer, poly(caprolactone) (PCL), polyhydroxybutyrate/valerate copolymer, poly(ortho ester), and poly(ethylene oxide) (PEO)/poly(butylene terephthalate) as coaters of Wiktor stent was studied for the implantation in animal models (Hårdhammar et al. 1996). The results, however, were not satisfactory to the group and were failed. The primitive clinical trial for biodegrdable failed due to the adverse effects of thrombosis, moderate intimal hyperplasia, and inflammatory response. Lack of manufacturing the engineered polymer for inhibiting inflammation and restenosis led the project to this failure (Garg and Serruys 2010; Van Der Giessen et al. 1996). A fundamental prerequisite for long-term survival of biomaterials is that these materials are compatible with physiological condition, i.e., a successful healing process necessitates not only utilizing biomaterials that is compatible with the host immune system, but also having a profound understanding of the host immune response towards implanted biomaterials (Franz et al. 2011). Later, Lincoff et al. (1997) took advantage of high-molecular weight poly(l-lactide) (PLLA) (321 kDa) for stent coating, and they compared the results with the low-molecular-weight (80 kDa) in a porcine model. The results proved the intense inflammatory neointimal hyperplasia within low-molecular-weight PLLA, while no major adverse effect was reported with high-molecular-weight PLLA. Yamawaki et al. (1998) reported the first successful outcome from the animal model. The contribution of permanent polymer coating in increasing the adverse effects of DES implantation such as delayed healing, late ST, local hypersensitivity reaction, and ISR was confirmed by Busch et al. (2014). Two biostable polymers used for drug-eluting stents, namely PEVA and PBMA, and four biodegradable polymers from the polyesters group, namely PLLA, poly(3-hydroxybutyrate), poly(4-hydroxybutyrate) (P(4HB)), and a polymeric blend of PLLA/P(4HB) in a ratio of 78/22% (w/w) were chosen for comparison. The material-dependent endothelialization, SMC growth, and thrombogenicity were proved by in vitro tests conducted through cultivating human umbilical venous endothelial cells, human coronary arterial endothelial cells, and human coronary arterial SMCs on the surface of these polymers (Busch et al. 2014). Biodegradation implies the dispersion of polymeric materials as a consequence of macromolecular degradation (Generali et al. 2014; Vert 1989; Vert et al. 1992). Degradation is to be used for those ex vivo mechanisms, whereas biodegradation is restricted to all in vivo mechanism mediated in cells (Onuma and Serruys 2011; Vert 2009). PLA and PGA are of two most ubiquitous polymers that have been exploited in the second-generation DES. Huang et al. (2010) reported promising clinical results on their patented stent (Venkatraman et al. 2008). This is a dual drug-eluting stent (DDES) with two pharmaceutical drugs: one acts as an anti-proliferative (sirolimus) and the other acts as an anti-thrombotic (triflusal). Drugs were loaded in the biodegradable polymer matrix contributing together as coating layers on a CoCr stent. In this study, poly(d,l-lactic-*co*-glycolic acid) (PDLGA) was employed as a drug carrier for its known properties of biocompatibility, biodegradability, and good mechanical strength (Huang et al. 2010). This 2-layered dual-drug-coated stent was prepared by spray coating a biodegradable polymer loaded with dual drugs on the metallic frame. The presence of triflusal as a drug agent in the polymer accelerated the polymer breakdown. The in vivo results showed promising results from the combination of two drugs on the stent surface in comparison to a bare metal stent, a sirolimus-coated polymer-coated stent, and a pure polymer-coated stent (controls) (Huang et al. 2010). Problems with using polymers as an outer layer of stent have been reported as well. First, the expansion of the stent during deployment may expose the polymer on the outer layer to great amounts of stress that leads to mechanical damage such as cracks, waviness, depressions, and peeling as reported by others (Basalus et al. 2009; Basalus et al. 2012; Otsuka et al. 2007; Wiemer et al. 2010). Second, there might be inflammatory and hypersensitivity reactions of body immune system to some polymers. Biodegradation does not necessarily mean that no sensitivity would not happen as van Beuskeom et al. (2000) have found. They reported the extensive inflammatory responses of these polymers and their fibrocellular proliferation except for poly(metacryloyl phosphorylcholine lauryl methacrylate). Further in a study by van der Giessen et al. (1992), they undertook a comparative study for the rate of thrombosis and neointimal formation in synthetic biodegradable poly (methyl methacrylate) coated and uncoated stainless steel-based stents in animal models (Guildford et al. 2010). Finally, polymers may delay the growth of vascular endothelial cells (Lancaster et al. 2012); all disadvantages that further led to the idea of polymer-free stents (Hu et al. 2015). Further improvement on the performance of DESs will require the well-selected polymer coating matrices with the properties of degradability and low inflammatory response during polymer degradation to allow a quick and complete DES endothelialization (Guildford et al. 2010).

Biodegradation consists of bulk-erosion and surface erosion (Langer 1995). Degradable polymers with surface erosion tend to be eroded quickly at the surface without the penetration of water molecules through hydrolysis. Some examples of this type of polymer includes poly(carboxyphenoxy hexane-sebacic acid) (Anastasiou and Uhrich 2000; Staubli et al. 1991; Tamada and Langer 1992), poly(fumaric acid-sebacic acid), poly(carboxyphenoxy hexane-sebacic acid), poly(imide-sebacic acid), poly(imide-carboxyphenoxy hexane), and poly(ortho ester), to name but a few (Schwach-Abdellaoui et al. 1999). In contrast to surface erosion, bulk erosion polymers are hydrophilic with water molecules’ absorption and hydrolysis occurs uniformly across the polymer matrix. Some examples of bulk erosion polymers are poly(α-hydroxy esters) such as PLA, PGA, and poly(lactic acid-*co*-glycolic acid) (PLGA), and also PCL, poly(*p*-dioxanone), poly(trimethylene carbonate), poly(oxaesters), poly(oxaamides), and their co-polymers and blends (Middleton and Tipton 1998). Other bulk erosion polymers are tyrosine-derived poly amino acid (Ertel and Kohn 1994; Fiordeliso et al. 1994; Pulapura and Kohn 1992; Pulapura et al. 1990) such as poly(DTH carbonates), poly(arylates), and poly(imino-carbonates), phosphorous-containing polymers such as poly(phospho-esters) and poly(phosphazenes) (Andrianov et al. 1994; Ibim et al. 1997; Lemmouchi et al. 1998), poly(ethylene glycol) (PEG)-based block co-polymers [PEG-poly(propylene glycol), PEG-poly(butylene terephthalate)], poly(α-malic acid), poly(ester amide), and poly(alkanoates) such as poly(hydroxybutyrate) and poly(hydroxyvalerate) copolymers (Parker et al. 2010). Polymers can constitute the base scaffold and (or) the coating of a stent. The polymeric coating layer allows the drug to have a gradual release in a time frame (Babapulle and Eisenberg 2002a; Babapulle and Eisenberg 2002b; Dang et al. 2014; Regar et al. 2001). In Table 1 a list of biodegradable polymer coating DES is prepared with a list of drugs to be loaded in the stent. Despite positive results reported from polymer coating stents, recently, there have been great efforts for a fully polymeric drug-eluting stent. Their main limitation, however, is their under-expected mechanical properties for clinical applications (Dang et al. 2014).

**Table 1:** Drug-eluting stents with biodegradable polymer coating

	Stent	Manufacturer	Stent material	Polymer	Coating method	Drug	References
1	BioMatrix	Biosensors	Stainless steel	PLA	Abluminal: Biolimus A9 + PLA	Biolimus A9	Muramatsu et al. (2013)
2	eucaTAX	Eucatech	Stainless steel	PLGA	–	Paclitaxel	Grabo et al. (2010)
3	Infinnium	Sahajanand	Stainless steel	PDLLA-co-PGA, PNVP, PLLA-co-PCL	The drug is coated in 3 different layers of combination of drug and polymer, and each layer has a different release profile.	Paclitaxel	Garg and Serruys (2010)
4	Luc-Chopin	Balton	Stainless steel	PLGA	Palitaxel + PLGA	Paclitaxel	Graboet al. (2010)
5	Nobori	Terumo	Stainless steel	PLA	Abluminal: Biolimus A9 + PLA	Biolimus A9	Garg and Serruys (2010)
6	JACTAX	Boston Scientific	Stainless steel	PLA	Abluminal (have 2750 discrete microdot): paclitaxel + PLA	Paclitaxel	Garg and Serruys (2010)
7	Sparrow	CardioMind	Nitinol	PLLA, PLGA, PLC, PVP	Sirolimus drug + polymer matrix	Sirolimus	Garg and Serruys (2010)
8	Supralimus	Sahajanand	Stainless steel	PLLA, PLGA, PLC, PVP	The layer: sirolimus + PLLA, PLGA, PLC; the outer: PVP	Sirolimus	Muramatsu et al. (2013)
9	BioMime	Meril Life Science	Co-Cr	PLLA + PLGA	–	Sirolimus	Muramatsu et al. (2013)
10	Excel	JW Medical System	Stainless steel	PLA	Abluminal: sirolimus + PLA	Sirolimus	Muramatsu et al. (2013)
11	Axxess	Biosensors Europe SA	Stainless steel	PLA	Abluminal: Biolimus A9 + PLA	Biolimus A9	Muramatsu et al. (2013)
12	Orsiro	Biotronik AG	Co-Cr	Abluminal side: PLLA; luminal side: silicon carbide layer	Abluminal: the Biolute polymer: PLLA and sirolimus; luminal: amorphous hydrogen rich silicon carbide	Sirolimus	Muramatsu et al. (2013)
13	MAHOROBA	Kaneka	Co-Cr	PLGA	Rollcoat abluminal: tacrolimus + PLGA	Tacrolimus	Hu et al. (2015)
14	Synergy	Boston Scientific	Co-Cr	PLGA	Abluminal: everolimus + PLGA	Everolimus	Garg and Serruys (2010)
15	NOYA	Medfavor Medical	Co-Cr	PDLLA	Sirolimus + PDLLA	Sirolimus	Muramatsu et al. (2013)
16	Combostent	OrbusNeich Medical	Stainless steel	SynBiosys	Abluminal surface: sirolimus + SynBiosys; luminal: CD34 antibody layer	Sirolimus	Garg and Serruys (2010)
17	Inspiron	Scitech Medical	Co-Cr	PLLA + PDLLGA	Abluminal: sirolimus + PLLA + PDLLGA	Sirolimus	Hu et al. (2015)
18	TIVOLI	Essen Technology	Co-Cr	PLGA	Sirolimus + PLGA	Sirolimus	Muramatsu et al. (2013)
19	BuMA	SinoMed	Stainless steel	PLGA	Abluminal: base layer: poly(n-butyl methacrylate); drug layer: sirolimus + PLGA	Sirolimus	Muramatsu et al. (2013)
20	Firehawk stent	MicroPort Medical	Co-Cr	PDLLA	An abluminal groove: sirolimus + PDLLA; luminal: PDLLA	Sirolimus	Muramatsu et al. (2013)
21	Conor	Conor Medsystems	Stainless steel	PLGA	Reservoirs: PLGA and paclitaxel	Paclitaxel	Hu et al. (2015)
22	Cardiomind	Cardiomind	Nitinol	PLA + PLGA	–	Sirolimus	Guildford et al. (2010)
23	Champion	Boston Scientific	Stainless steel	PLA	–	Everolimus	Guildford et al. (2010)
24	Symbio	Cordis	Cobalt-Chromium	PLGA	–	Pimecrolimus + paclitaxel	Guildford et al. (2010)

### Biodegradable scaffolds

**Fig. 1:**
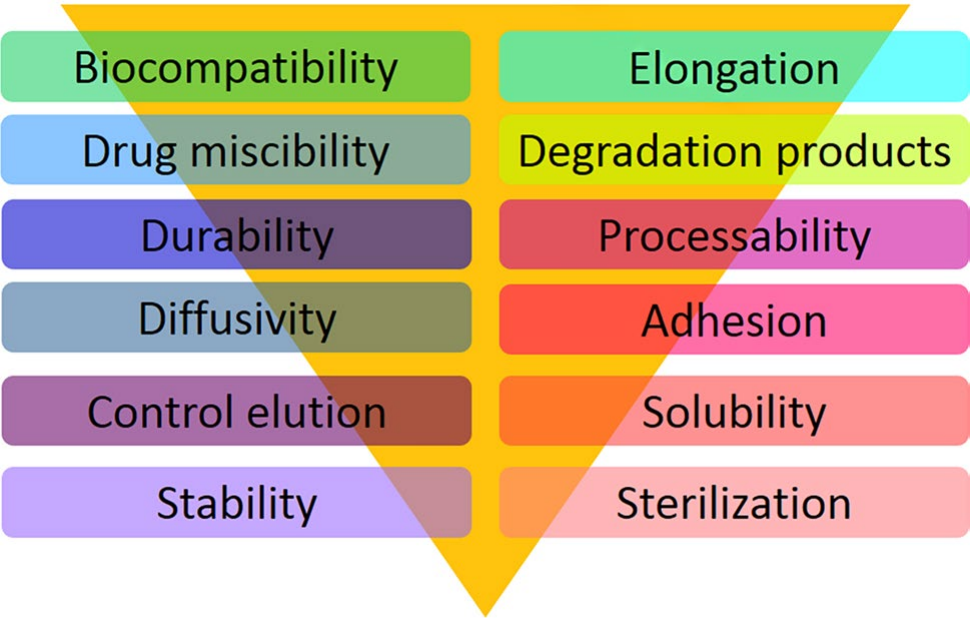
Requirements for biodegradable polymer-based drug-eluting stents

Loading drugs on first-generation DES was achieved through polymer coating on the stent surface. Polymers were considered to initiate inflammatory response contributing to in-stent restenosis (ISR). To address this problem, new polymer materials with enhanced biocompatibility and biodegradability were used for stent backbone. These stents with thinner struts are known as second-generation DESs. Despite improved safety of the implant, this type of stent had still a permanent backbone (Naseem et al. 2017). An alternative replacement which is still under research and development is a new generation of stents with bioresorbable scaffolds which gives a temporary support to the artery and fully biodegrades after its complete functionality. Scaffolds, as stated in papers, play a key role in vascular restoration therapy associated with endothelial function and vasomotion (Celermajer 1997; Oberhauser et al. 2009; Naseem et al. 2017). Functional endothelial coverage contributes to a reduced in-stent thrombosis rate, which is a prerequisite for long-term use of anti-platelet therapy (Ormiston and Serruys 2009). In a perfectly ideal condition, after completing the stent’s function it is desirable for the stent to be dissolved and let the artery revert to its normal condition. Fully degradable stent not only allows the artery to revitalize, but also it makes any other re-intervention or treatment to the affected site easier (Driver 2012). Bioresorbable cardiovascular scaffold (BCS) is a propitious alternative to permanent stents. It has been termed the fourth revolution in international cardiology (Wayangankar and Ellis 2015). The term scaffold indicates the temporary nature of BRS which is in opposition to the permanent implant (Wiebe et al.^2014^). The properties of an ideal biodegradable scaffold are shown in Table 2. Polymers utilized in BRSs should pass a bioresorption process rather than bioabsorption in order to lessen the adverse effects of body defense reactions to the least possibility. As Vert et al. (Vert 1989; Vert et al. 1992) reported earlier the degradation products of polymer will not completely be eliminated from the site of action and remain inside the human body which defines bioabsorption. Bioabsorption reflects the ability of polymeric materials and devices to dissolve into human body without breakdown of the polymeric chain or reduction in molecular mass (Generali et al. 2014; Vert 1989; Vert et al. 1992). In spite of bioabsorption, during the bioresorption process, degradation breaks the polymeric chains down into low-molecular-weight compounds. These remnants will mostly engage in metabolic cycle to be eliminated from the site of action by disposing through kidneys or lungs. As it clearly appears, there is a subtle difference between bioabsorption and bioresorption, two of which are sometimes interchangeably used (Onuma and Serruys 2011). In this article, the term biodegradation is substituted for two terms of bioresorption and bioabsorption to avoid complexity and confusion of terms. In Fig. 1 the requirements of bioresorbable polymer-based DES are listed. Overall, applying DES has proven to be one of the safest method for inhibiting acute recoil, negative remodeling, and neointimal hyperplasia (Al Suwaidi et al. 2000; Nobuyoshi et al. 1988; Serruys et al. 1994; Sigwart et al. 1988; Stone et al. 2005).

**Table 2:** Properties of an ideal biodegradable scaffold (Wayangankar and Ellis 2015; Mariano et al. 2013)

Biocompatibility: before, during and after degradation
Adequate radial strength
Adequate time for degradation; not too fast to increase inflammation, and not too long to provoke adverse body reaction, 4–6 months
No inflammatory process aggravating or initiating by degradation
Compatibility with DES technology and eluting drugs at a determined rate without any effect on the radial strength
Not having thick struts
Easy deliverability
Easy refrigeration
Enhanced visualization under fluoroscopy
Compatible with currently available equipment for deployment
Improved dwell time before deployment

Working with permanent metallic stents has its own complication, especially with respect to repeat interventions. Conventional metal stents are present through the life time of the patient and would not degrade after a short period of time; therefore, for those patients who need to receive additional metal stents in downstream lesions there would be a problem in passing a new stent through the already implanted stent (Kohn and Zeltinger 2005). The absence of a permanent scaffold has the potential to overcome the shortcomings of the conventional BMS or metal-based DES (Wayangankar and Ellis 2015). A biodegradable scaffold is subject to a transient framework, i.e., the biodegradable stent would maintain the vessel open until the end of its mission; in the next step, the stent would disappear from the site by bioresorption. This type of stent allows the vessel to return to its initially natural state without any blockage (Bourantas et al. 2013). Some highlights about BRSs which make this technique superior to BMSs and DESs are adaptive shear stress, late luminal gain, late expansive remodeling, reduction in restenosis and late stent thrombosis, re-intervention possibility at the site of injury, and improved invasive imaging. Two predominant advantages about BRSs are capacity to restore natural vascular function, and higher flexibility in comparison to metal backbones. All these bright sides plus the two of the above-mentioned highlights could change the outcomes of cardiovascular interventions (Naseem et al. 2017).

Expansion of the stent in an artery exerts stress to the wall of the site. But the stress if not managed could be high enough to hurt the vessel wall and put the way to restenosis. The pressure on the inner side of the artery makes the tissue between the struts take the form of the stent. It is suggested that the deflection of the tissue being in contact with the stent structure (prolapse or draping) is a rough estimation for the potential of a stent to cause restenosis (Prendergast et al. 2003). Another difficulty with the rigid metallic frame is disturbing the flow within the vessel altering the pulsatile profile. Having the flexibility to allow the vessel respond to any variations in the profile of pulsatile flow is highly important due to the effect of implantation on the geometry of curved vessels. It affects the shear stress distribution and can be regarded as a risk factor for neointima formation (Bourantas et al. 2013; Gijsen et al. 2003; Gomez-Lara et al. 2010; Orr et al. 2010; Serruys et al. 2011a, b; Tortoriello and Pedrizzetti 2004; VORPAHL et al. 2009). Normally, the rigid body of a metallic stent cannot allow the vessel do the normal function. Gyöngyösi et al. (2000) have proven all adverse effects made by metallic stents regarding the fact that higher inflammation and other related cascade events are all the results of the straightening of the artery by metallic stents. Curvature and angulation are two important terms with respect to the modification of vessel geometry as a post-deployment effect (Wayangankar and Ellis 2015). Gomez-Lara et al. (2010) studied curvature and angulation of treated vessels after deployment of both a metallic stent and a bioresorbable vascular stent to analyze the conformability of the stent in the vessel. They found that conformability is a determinant parameter for geometric changes in coronary arteries. Further studies also confirmed that BRS implants make the implanted artery revert to their previous geometry within 6–12 months of implantation over previous generations (Gomez-Lara et al. 2011; Wayangankar and Ellis 2015). A study (Wayangankar and Ellis 2015) showed that BRSs outperformed those DES stents. As stated in a paper by Brugaletta et al. (2012) the BRS provides favorable vascular dynamics with minimal shear stress and late lumen enlargement: two of factors attributed to future cardiovascular events (Wayangankar and Ellis 2015). Elsewhere, the implantation of everolimus-eluting bioresorbable vascular scaffold (BVS) (Abbott Vascular, Santa Clara, California) resulted in a modest change in vessel curvature and angulation (Gomez-Lara et al. 2010). The changes have been more observed for vessels with severe baseline curvature and angulation. In comparison to conventional metallic platforms, BVSs implied better conformability and in regard to their functionality they outperformed (Gomez-Lara et al. 2010). A case study of 58 patients within the ABSORB^1^ 1.1 trial (Brugaletta et al. 2012) has concluded that the symmetry of neointimal thickness was higher at 12 months when compared to 6 months indicating treated vessels reverted to the pre-stenting geometry (Wayangankar and Ellis 2015). Despite all advantages, there are drawbacks within the polymeric backbone of stents as follows: (1) the lack of radio-opacity which is necessary for the precise placement and monitoring the stent location within the vessel. A stent needs to be visible by X-ray radiography/fluoroscopy. Since the use of MRI imaging is increasing as the common method of tracing, stents must also be visible by MRI. The metal stents have the advantage of being visible in contrast to degradable polymers that need metal marker at the distal part of the stent, or covering the surface with a metal coating, but none of these two options were proven to be satisfactory. There are recently new polymers that have been found to be inherently radio-opaque which are based on the iodination of the tyrosine ring in tyrosine-derived polycarbonates. These polymers can be clinically applicable for X-ray imaging and MRI (Kohn and Zeltinger 2005). (2) Reduced radial strength compared to their metallic counterparts. Although metal stents are much tougher and stronger than degradable stents, this lower mechanical strength of degradable DESs does not exert excessive amounts of stress to the vessel wall, which in case could adversely affect vessel perfusion and healing of the vessel (Palmaz 1993; Rab et al. 1991; Yang et al. 1991). During degradation of a DES this stress is even alleviated until the complete disappearance of the stent. There are certain mechanical prerequisites for a stent to be implanted in coronary arteries including high-elastic moduli for increased amount of radial stiffness, large-break strains to give the stent the ability of withstanding deformations from the crimped to the expanded state, and low-yield strains to alleviate the amount of recoil (Kohn and Zeltinger 2005). The collapse of the stents into small pieces due to failing of mechanical strength will exacerbate the problem by blocking the vessel rather than the presumed functionality of re-opening it. Hence, there are both good points and bad points in each of biodegradable and non-biodegradable materials (Waksman 2006). (3) Reduced flexibility of the stent (Waksman 2006). Bioresorbable scaffolds are still at their preliminary level of experimental and computational analysis, and furthermore clinical trials are needed to conduct to analyze their efficiency in human-simulated environment (Naseem et al. 2017). To make a polymeric stent prepared to have suitable mechanical properties, there is a great demand to increase strut dimensions. In this way, there is a make up for mechanical shortcomings of a biodegradable scaffold (Onuma and Serruys 2011). Increased thickness in struts to compensate for reduced mechanical strength leads to unfavorable events such as vessel injury, non-laminar flow within the stent, making the stent into a favorable scaffold for platelet deposition and a diligent implantation (Guildford et al. 2010). It is reported that the following three polymers have the highest mechanical strength comparing to the rest of polymer materials: high-molecular-weight poly*(*l-lactic acid) (Tepha, Inc.), silk-elastin polymers (Protein Polymer Technologies, Inc.), and tyrosine-derived polycarbonates (Rutgers University and REVA Medical, Inc.) (Kohn and Zeltinger 2005). (4) During degradation of some polymer-based stents such as PLGA-based stent, the significant pH change of the medium due to the acid-nature of the polymer could lead to the necrosis of the cells in contact (Kotsar et al. 2008). (5) The longer time of pre-dilatation. For a stent to be implanted, it is mandatory to pre-dilate the site of injury with a longer balloon, which is inflated after its deliverability to the vessel. For BRSs, due to insufficient radial strength and diligent deliverability especially in complex lesions, prolonged and time-consuming pre-dilatation is required compared to conventional stents. The longer time of pre-dilatation increases the risk of stent fracture and this possibility makes researchers to oversize the stent struts or increase the time of inflation compared to the normal time of pre-dilatation (Wiebe et al. 2014). Other complementary explanations about BRSs are listed in Table 3. All these dexterity and assiduousness with BRS handling lead to higher costs and duration of percutaneous coronary intervention compared to conventional DES (Wiebe et al. 2014).

**Table 3:** Potential advantages and disadvantages of biodegradable-based stents (Bourantas et al. 2013; Kereiakes et al. 2016; Onuma and Serruys 2011; Sharkawi et al. 2007)

Potential advantages	
Restoration of cyclic pulsatility and normal vasomotion	Prevented acute occlusion
Prevented acute ST and subacute ST	Restoration of normal vessel curvature
Normalizing shear stress and cyclic strain	Prevented acute recoil
Prevented constrictive remodeling	Prevented expansive remodeling
Reduced risk of very late polymer reactions	Avoidance of stent malapposition
Reduced neoatherosclerosis	Avoidance of late luminal enlargement
Avoidance of late vessel wall inflammation	Prevented neointimal hyperplasia
Prevented late ST	Formation of a cap over lipid-rich plaque
Unjailing of side branches	
Disadvantages	
Unsuitable release profile for drug delivery system	Difficulty in delivery to the site of action because of thicker struts with larger crossing profile
Greater risk of acute strut fracture as a result of insufficient mechanical strength compared with metallic DES	Inadequate degradation and resorption profile
Increased rates of early thrombosis	Inflammatory degradation residues
Specific (cold) storage condition and specific deployment techniques	

#### How does a bioresorbable scaffold function?

**Fig. 2:**
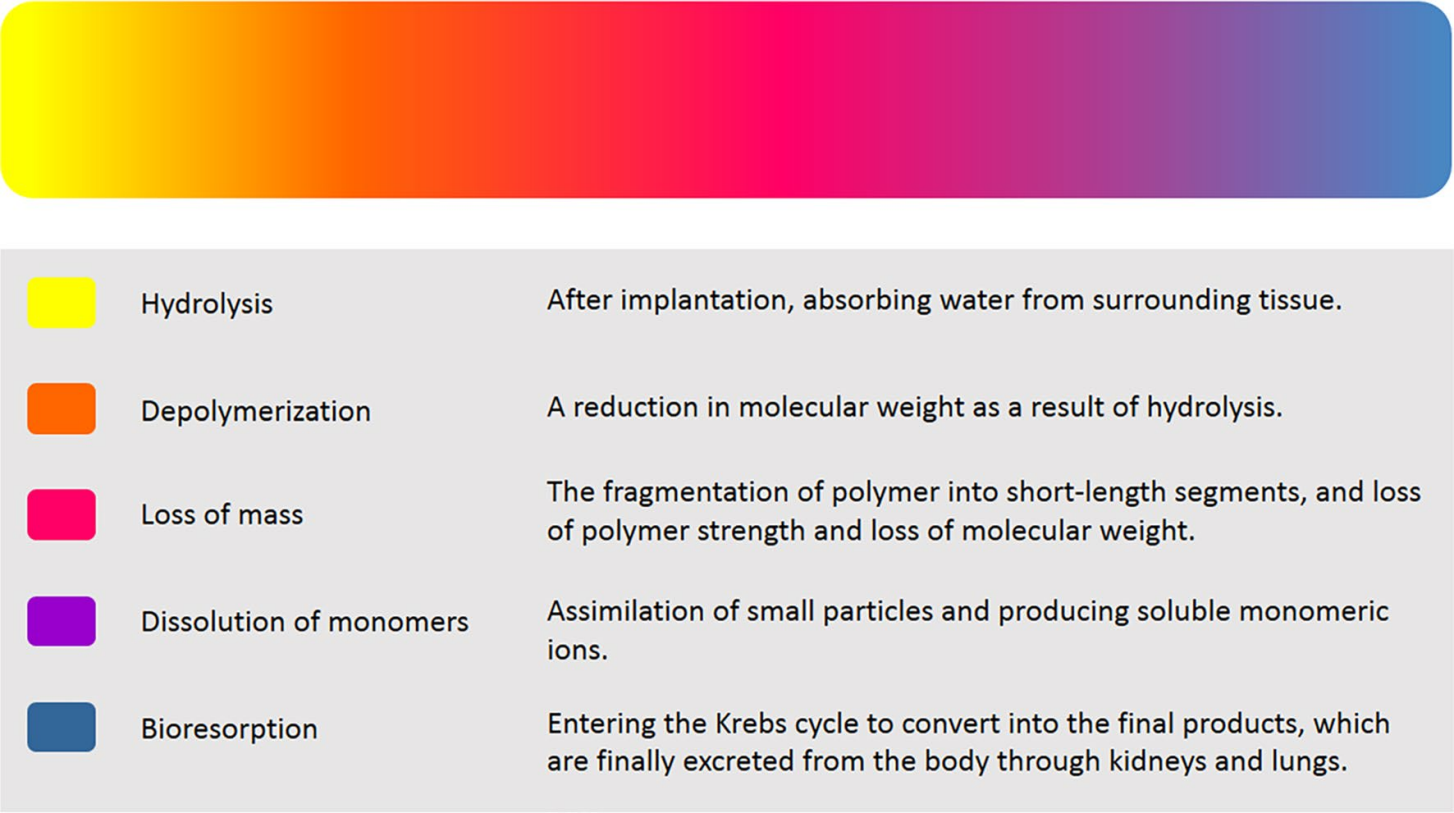
Phases of biodegradation for bioresorbable scaffolds (Onuma and Serruys 2011)

**Fig. 3:**
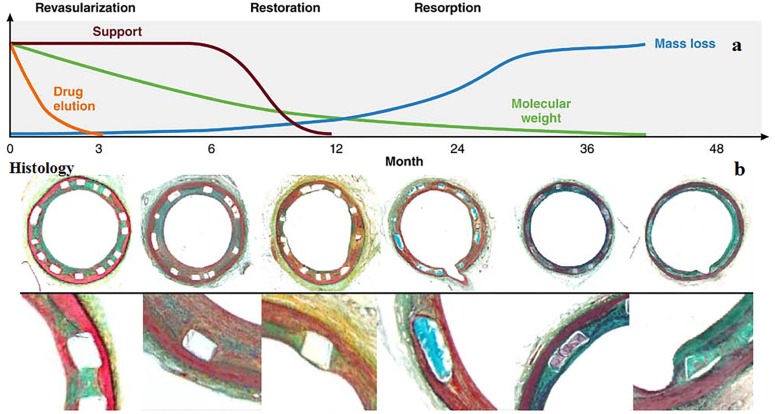
The three phases of BRS functionality include mechanical support and drug delivery functions during the revascularization phase; the loss of radial rigidity and mechanical restraint during the restoration phase, during which cyclic pulsatility and vasomotion return; and resorption caused by mass loss with return of adaptive vascular remodeling responses. The time course for phases/changes noted on the top is specific for the absorb bioresorbable vascular scaffold. Molecular weight starts to decrease immediately after implantation, and drug elution is almost completed at 3 months. Radial support decreases at ~ 6 months and is minimal at 12 months. Representative histology images are from Yucatan swine. At 24 months, with progressive mass loss, the strut footprints begin to be replaced by provisional matrix (b-histology). At 36 months, mass loss is completed, and infiltration of connective tissue into strut voids makes the struts invisible on OCT between 36 and 48 months (Kereiakes et al. 2016)

There are three overlapping phases of functionality for a BRS, namely revascularization, restoration, and resorption (Fig. 2). Revascularization deals with the problem of narrowing vessels to re-open them. Greater flexibility and conformability of the biodegradable polymer with the vessel geometry and the superiority in maintaining the normal vessel curvature make it a good candidate as an alternative for BMSs and metal-based DESs. In Table 4, an overall pairwise comparison supplies readers with informative comparison. Restoration is the second phase for the full functionality of BRS. In this phase, there is a loss in total mass of the molecule which emerges in the reduction of molecular weight. Hydrolysis and depolymerization followed by metabolism of the initial production of lactate into carbon dioxide and water. The degradation process leads to the weight loss of the polymer structure. The last and the third phase, resorption, is the complementary phase for full recovery of vascular structure to its initially normal function. The three phases of BRS functionality are integrally brought together in Fig. 3a (Kereiakes et al. 2016). To be more elaborate, these three phases embrace five overlapping stages of degradation inside consisting of hydrolysis, depolymerization, a loss of mass, dissolution of the monomer, and bioresorption (Onuma and Serruys 2011). The incidence of resorption is evident by taking serial intravascular ultrasound and optical coherence tomography (OCT) imaging in patients. Imaging technique (Fig. 3b) has revealed that within 12–18 months after BRS deployment the vessel lumen areas have enlargement (Kereiakes et al. 2016). The opposite incidence takes place within metallic DES with mean lumen diameters decrease over time and these decrease stems back to the growth of plaque in a permanent metal platform (Kereiakes et al. 2016; Kimura 2015). The most widely used polymers for the design of degradable stents are PLLA, PGA, PCL, and their copolymers (Abizaid et al. 2015; Capodanno et al. 2015; Puricel et al. 2015). Aliphatic polymers and copolymers are suggested as attractive biomaterials in BCSs for human implantation. There are advantages over applying these polymers: first, as studies indicated aliphatic polymers and their copolymers are beneficial for vascular tissue recovery. Second, the final degradation products of these polymers, CO_2_and H_2_O, can be eliminated from the human body through natural metabolism. Despite favorable advantages, there are drawbacks which follow: first, the intermediate degradation products reduce local pH and cause inflammation. Second, these polymers have a slow expansion in the blood vessel. Third, their implantation in the coronary arteries require heating of the balloon that is unfavorable to the blood vessel and could lead to serious injuries (Jiang et al. 2017). There are also two major concerns over using these degradable polymers: first, they are not visible by fluoroscopy, and last, not sufficient mechanical properties (Kereiakes et al. 2016).

**Table 4:** Pairwise comparisons of BA, BMS, DES, and BVS

	Advantages	Disadvantages	References
BA (Balloon angioplasty)	Widen a blocked vessel	Acute vessel closure, elastic recoil, neointimal proliferation, late constrictive remodeling	Simard et al. (2014), Hara et al. (2006), Iqbal et al. (2013), Naseem et al. (2017), Pourdjabbar et al. (2011)
BMS VS. BA	Declined rate of restenosis incidences, ability to maintain a widened vessel in long-term	Thrombosis, neointimal hyperplasia, and chronic inflammation	Rensing et al. (2001), Jiang et al. (2017), Serruys et al. (2006), Serruys et al. (1994), Schatz et al. (1991)
Nondegarable-based DES (traditional DES) VS. BMS	Treating hyperplasia and inflammation temporarily, minimized smooth-muscle proliferation (neointimal hyperplasia), reduced restenosis rate and decreased rates of target lesion revascularization by 50–70%	Long-term presence of non-degradable biomaterials in the vessels leads to thrombosis, neointimal hyperplasia, and chronic inflammation	Serruys et al. (2006), Kočka et al. (2015), Gada et al. (2013)
Biodegradable-based DES VS. BMS	Minimized SMC proliferation, reduced restenosis rates, reduced rates of target lesion revascularization	Inflammatory response, late stent thrombosis, delayed healing, Immunosuppressive, the drugs loaded on DES delayed vascular healing and re-endothelialization, high risk of late thrombosis	Kočka et al. (2015), Serruys et al. (2006), Qureshi and Caplan (2014), Yang et al. (2014)
BRS VS. permanent stenting BMS	Enhanced arterial recovery, positive remodeling, restore natural vascular response in the vessel, significant reduction in restenosis and late stent thrombosis, a potential for re-intervention, higher flexibility	Bioresorbable polymers are prone to stress relaxation, there is still a lack of works on evaluating both the pre-degradation properties and degradation performance of these scaffolds, their use in clinical procedures is still sparse due to the lack of experimental and computational analysis of their efficiency and suitability, with elastic recoil, constrictive remodeling and endothelium dysfunction being of concern	Serruys et al. (2009), Ormiston et al. (2007), Iqbal et al. (2013), Serruys et al. (2011b), Naseem et al. (2017)

#### Materials of the BRS

##### Poly-l-lactic acid (PLLA)

**Fig. 4:**
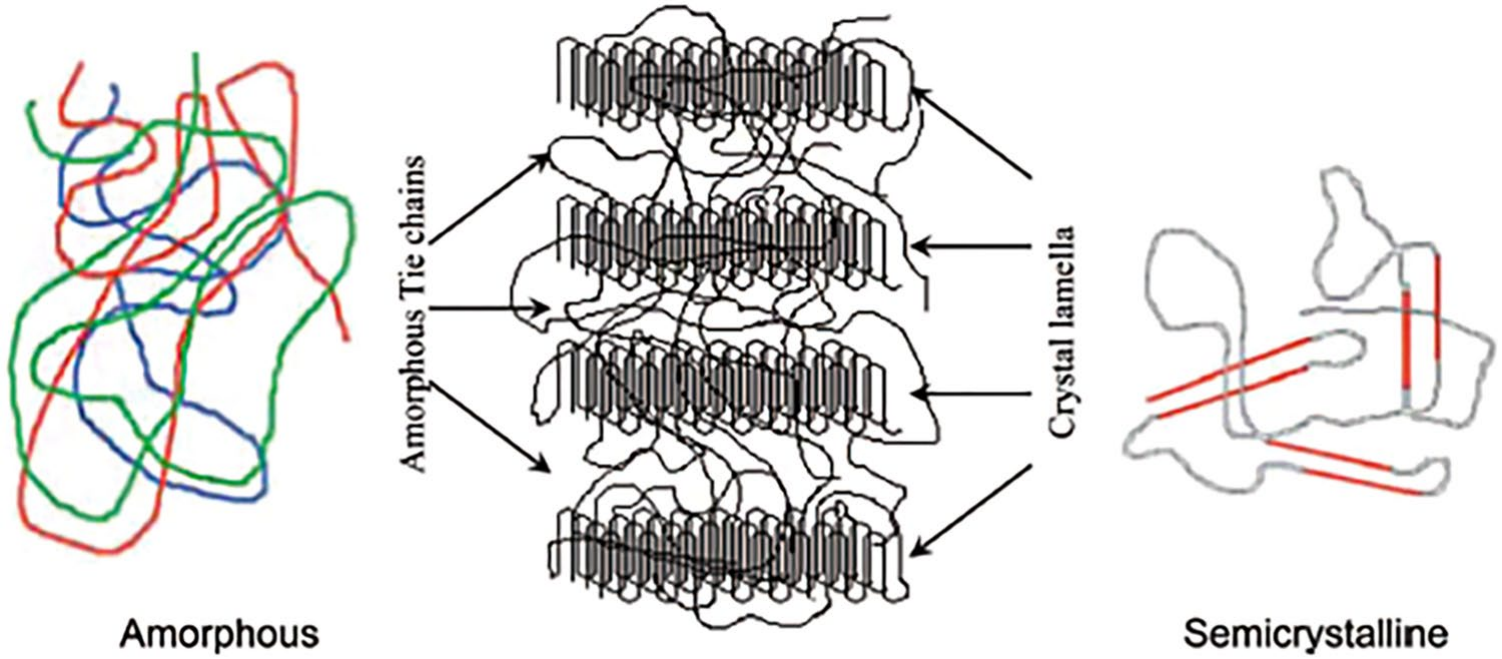
Schematic presentation of amorphous polymer (left), semi-crystalline structure of the PLLA with crystal lamella (crystalline polylactide) interconnected by amorphous tie chains binding the lamellae together (middle) and semi-crystalline polymer (right) (Onuma and Serruys 2011)

A BRS is exposed to huge amount of stress and strain during crimp and expansion due to implantation process in the body. In regard to degradable polymers, PLLA of different molecular weights is the base material of vascular and cardiovascular stents. Polylactic acid is a popular polymer for medical applications and is the first polymer-based scaffold which got the FDA approval in 2016 (Naseem et al. 2017). The main reason for PLLA to be the most commonly used material for biodegradable scaffolds is its unique mechanical strength towards high load cycles, i.e., after the deployment of a stent, there is a great demand for maintaining the mechanical integrity (Bergström and Hayman 2016; Eyring 1936; Hayman et al.^2014^; Nakafuku and Takehisa 2004). The end product is lactic acid, which in turn is metabolized into carbon dioxide and water through Krebs cycle (Ormiston et al. 2008). It is assumed that complete degradation is achieved in 1–3 years (Wiebe et al.^2014^). An interesting point about PLLA is its molecular structure. It is a semi-crystalline polymer with the random or amorphous segments, which are distributed throughout the polymer structure between the ordered polymer chains known as crystal lamella. The crystallites as shown in Fig. 4 are interconnected by the random binding chains (Onuma and Serruys 2011). The PLLA-based stents have an admixture of semi-crystalline polymers and amorphous polymer. Crystallinity brings out mechanical strength to the system while the latter facilitates the dispersion of drug molecules in the polymer matrix. The amorphous segment determines the rate of degradation while crystal domain of the polymer determines the absorption rate (Onuma and Serruys 2011; Wayangankar and Ellis 2015). The degradation behavior of PLLA is of high interesting characteristic to medical applications. The degradation rate is controlled by the following factors: the molecular weight and orientations, the degree of crystallinity, the applied load (Van Dijk et al. 2002; Zhou et al. 2010). The degree of crystallinity is important for the degradation rate of the polymer. The crystalline domains within a polymer have low tendency to water molecules, which in turn brings about a slower rate of degradation as a result of slower hydrolysis (Vieira et al. 2011). Considering the magnitude of the applied stress, the rate of degradation will be affected. During degradation, mechanical factors consisting of yield stress, yield strain, and elongation at break decrease significantly (Grijpma and Pennings 1994).

Despite their dominance as the contributing stent material, there are still uncertainties remaining with this polymer which needs further analysis involving first, understanding mechanical behavior during and after implantation in the site of injury and during its degradation. Second, comparison the performance of the PLLA-based stent and metal-based stents (Naseem et al. 2017). There are complications within the application of PLLA as the base material. The lower stiffness and strength of PLLA materials make stent struts to be thicker in comparison to conventional metal-based stents. The increase in diameters of struts may lead to complications within the stent such as platelet adhesion, and vessel injury. Another complication is about the premature failure of the PLLA at the stress magnitude below the considered yield and the tensile strength of the material. The result is that long before the PLLA is degraded, the device fails in face of the liquid pressure and the exerted pressure from the vessel wall. The mechanical behavior of the PLLA and other bioresorbable polymers is non-linear greatly due to some factors including molecular weight, temperature, molecular orientation, crystallinity of the polymer, and degradation characteristics. So, it is of high importance to the manufacturer to be familiar with the polymer behavior under these conditions before applying the device in the human body. Analyzing the stent behavior under simulated body condition could best help the researchers in this area decide for the best choice of material and its composition (Bergström and Hayman 2016). Assessing the polymer features contributing to the stenting function would be possible by experimental analysis accompanied by analytical and numerical studies. In a review by Zhao et al., recent studies which dedicated on investigating the performance of PLLA-based stents over the past 15 years were overviewed. It covered a wide range of studies involving mechanical testing of PLLA material and PLLA-base scaffolds, and computational studies which have been opening a new perspective to the prediction of outcomes (Naseem et al. 2017).

##### Magnesium (Mg)

In addition to biodegradable polymers, biodegradable metals are considered as biovascular scaffolds. The metal that is utilized in metallic biodegradable stents must have both biocompatibility and biocorrosion, and magnesium has these two features together (Guildford et al. 2010). Due to biodegradability of magnesium (Mg) and its alloys, these metals have attracted a great deal of research as biomaterials in cardiovascular stents. The reaction between Mg and water molecules results in degradation of magnesium into Mg^2+^ ions and H_2_ molecules, shown as reaction 1 (Nguyen et al. 2013):

1 $${\text{Mg}}\left( {\text{s}} \right) + 2{\text{H}}_{2} {\text{O}}\left( {\text{l}} \right) \to {\text{Mg}}^{2 + } + 2{\text{OH}}^{ - } + {\text{H}}_{2} \left( {\text{g}} \right)$$

Degradation of Mg takes between 2 and 12 months depending on its composition. Recent studies have reported of 9–12 months of radial support for Mg-based stents (Wiebe et al. 2014). It has been demonstrated that magnesium stent implanted in an animal model lost its mechanical integrity in 35–36 days and no evidence of thrombosis was reported (Heublein et al. 2003). Not only magnesium, but also recent studies have reported on biodegradability, safety, and efficacy of iron-based stents. The animal model trials showed signs of degradation within 28 days after deployment. Through these days, no in-stent thrombosis, excess inflammation or even fibrin deposition was seen (Waksman et al. 2008). However, the success of this project needs long-term follow-up studies to analyze the efficacy of corrodible iron stent (Guildford et al. 2010). Studies over the possibility of using Mg and Mg alloys as implant materials have been started in 1878 by Witte et al. (2015) First-generation magnesium-based BRSs were non-eluting, i.e., they lacked anti-proliferative drug release from the stent surface; it was suggested that the emerging electronegative charge during degradation of the metal platform could be functioning as an efficient anti-thrombotic agent (Wiebe et al. 2014). A prototype coronary stent with magnesium-based alloy showed promising results (Heublein et al. 2003). The stent lost 50% of its weight within 6 months. In this study, local responses in coronary arteries were analyzed in a pig model as the in vivo test, and animal trials demonstrated positive results indicating thrombogenicity, biocompatibility, and tissue proliferation throughout certain time frames during the experiment (Heublein et al. 2003). There are also reports of utilizing magnesium-based biodegradable stents for clinical trials. In Table 5, there are some examples of Mg-based stents. Between these exampled stents, some of them are marketed and some, on the other hand, are still underdevelopment for future results. The biggest concern about Mg and its alloys is the rapid degradation (corrosion) which causes the failure of its applications for cardiovascular applications, and yet there is no successful report of Mg-based BCS in clinical trials (Haude et al. 2016a; b). To avoid it, a modified-surface coating needs to be developed to delay the degradation of the scaffold. Polymer coating has also been widely reported as an alternative solution to address this problem (Haude et al. 2016a; b; Jiang et al. 2017; Johnson et al. 2013; Li et al. 2010; Liu et al. 2017; Wong et al. 2010). Research work conducted by Jiang et al. in a recent study has confirmed the positive effect of biodegradable polymer coating on reducing Mg degradation according to their results. The results especially suggested further studies on PLGA as a potential biomaterial for cardiovascular applications (Jiang et al. 2017). There are two possible ways to improve the corrosion resistance of Mg and Mg alloys: (1) tailoring the composition and microstructure. (2) Surface treatment or a protective coating. It has been demonstrated that in most cases coatings increase the resistance of Mg and Mg alloys’ scaffold towards corrosion (Hornberger et al. 2012). Micro-arc oxidation (MAO) is a surface treatment method for improving the corrosion resistance of Mg-alloy (Lu et al. 2011). This technique was used for surface coating of a Mg-alloy-based stent. The coating consisted of the following layers: MAO/PLLA coating layer to improve corrosion resistance as well as control the Mg ions’ release. The other layer consisted of the combination of two layers; a layer of PLGA/PTX, and a layer of 100% PLGA with no PTX integrated together as a drug release layer. The coating films were coated on a Mg-alloy AZ81 stent for controlling the biocorrosion rate and drug release rate simultaneously. The drug release rate was in a linear-sustained manner with no burst release which is of high value to a drug-releasing system.

**Table 5:** BRSs with their properties

	Stent name	Manufacturer	Scaffold platform	Coating material
1	ABSORB BVS 1.0	Abbott Vascular, Santa Clara, CA, USA	PLLA	PLLA
2	BVS 1.1	Abbott Vascular, Santa Clara, CA, USA	PLLA	PLLA
3	ReSolve/ReZolve	REVA Medical, San Diego, CA, USA	Tyrosine-derived polycarbonate	Tyrosine poly carbonate with iodine
4	Fantom	REVA Medical, San Diego, CA, USA	Tyrosine-derived polycarbonate	Bioresorbable polymer
5	IDEAL (first generation)	Bioresorbable Therapeutics Inc., Menlo Park, CA, USA	The backbone consists of polylactide anhydride mixed with a polymer of salicylic acid with a sebacic acid linker	SA/AA
6	IDEAL (Second generation)	Xenogenics Corp; Canton, MA, USA	The backbone consists of polylactide anhydride mixed with a polymer of salicylic acid with a sebacic acid linker	SA/AA
7	Xinsorb	Huaan Biotechnology, Laiwu, China	PLLA	PDLLA + PLLA
8	DREAMS 1G	Biotronik, Berlin, Germany	WE43 alloy, 93% Mg and 7% rare earth elements	Mg alloy
9	DREAMS 2G	Biotronik, Berlin, Germany	WE43 alloy, 93% Mg and 7% rare earth elements	Mg alloy
10	ART 18AZ	Arterial Remodeling Tech., France	PDLLA	None
11	Fast	Boston Scientific, Natick, MA, USA	PLLA	PLGA
12	Igaki-Tamai stent	Kyoto Medical Planning Co, Ltd, Kyoto, Japan	PLLA	None
13	Amaranth	Amaranth Medical Inc., CA, USA	PLLA	PLLA
14	Fortitude	Amaranth Medical Inc., CA, USA	PLLA	Bioresorbable polymer
15	Acute BRS	OrbusNeich, Fort Laudedale, FL, USA	PLLA-based polymer	PLLA, l-lactic-co-ε-caprolactone, PDLA
16	AMS-1	Biotronik, Berlin, Germany	WE43 alloy, 93% Mg and 7% rare earth elements	None
17	AMS-2	Biotronik, Berlin, Germany	WE43 alloy, 93% Mg and 7% rare earth elements	None
18	AMS-3	Biotronik, Berlin, Germany	WE43 alloy, 93% Mg and 7% rare earth elements	None
19	DESolve	Elixir Medical Corp., CA, USA	PLLA	Matrix of polylactide-based polymer
20	Arterius	The Innovate UK (TSB), and University of Bradford, UK	PLLA with extrusion	PLLA
21	QualiMed	QualiMed Innovative Medizinprodukte GmbH, Winsen, Germany	Mg	PLLA
22	Mirage Microfiber Scaffold, Coronary Artery Scaffold	Manli Cardiology, Singapore	PLLA	PLLA
23	MERES 100	Meril Life Sciences, Mumbai, Maharashtra, India	PLLA	PLLA
24	REVA DES	REVA Medical, CA, USA	Polymer tyrosine-derived polycarbonate polymer	None

	Drug (concentration)	Drug release rate (days)	Strut thickness (µm)	Resorption time (months)	Radial support	References
1	Everolimus (8.2 µg/mm)	80% (28)	156	18–24	weeks	Onuma and Serruys (2011)
2	Everolimus	80% (28)	150	24–36	6 months	Ormiston et al. (2009)
3	Sirolimus	100% (> 30)	114–228	48	3–6 months	Hu et al. (2015)
4	Sirolimus (115 µg)	N/A	125	36	N/A	Kereiakes et al. (2016)
5	Sirolimus (8.3 µg/mm)	over 30 days	200	9–12	3 months	Bourantas et al. (2013)
6	Sirolimus (8.3 µg/mm)	over 30 days	175	9–12	3 months	Bourantas et al. (2013)
7	Sirolimus (8 µg/mm)	Ex vivo: 80% (28)	160	24–36	N/A	Bourantas et al. (2013)
8	Paclitaxel (0.07 µg/mm2)	N/A	120	9	3–6 months	Kereiakes et al. (2016)
9	Sirolimus (1.4 µg/mm2)	N/A	125	4–6	3–6 months	Kereiakes et al. (2016)
10	None	None	170	3–6	3–6 months	Bourantas et al. (2013)
11	Everolimus	100% (90)	100	12–24	N/A	Kereiakes et al. (2016)
12	None	None	170	24–36	6 months	Ormiston et al. (2009)
13	None	–	156	12–24		Bourantas et al. (2013)
14	Sirolimus	N/A	120	3–6	3–6 months	Kereiakes et al. (2016)
15	Abluminal side: sirolimus; luminal: CD34 + antibodies	–	150	–		Wiebe et al. (2014)
16	None	–	165	< 4	Days or weeks	Onuma and Serruys (2011)
17	None	–	125	> 4	Weeks	Onuma and Serruys (2011)
18	None	–	125	> 4	Weeks	Onuma and Serruys (2011)
19	Myolimus (3 µg/mm)	> 85% of the drug released over 4 weeks	150	12–24	3–4 months	Bourantas et al. (2013)
20	Sirolimus	N/A	N/A	N/A	N/A	Kereiakes et al. (2016)
21	Sirolimus	N/A	N/A	N/A	3 months	Kereiakes et al. (2016)
22	Sirolimus	N/A	125 for 3.0 mm and 150 for 3.5 mm diameters	14	N/A	Kereiakes et al. (2016)
23	Sirolimus (1.25 µg/mm2)	–	100	24	N/A	Kereiakes et al. (2016)
24	None	–	200	24	3–6 months	Ormiston et al. (2009)

## Cardiovascular stent design parameters

Stent design parameters can be listed as follows: the dimension of the stent struts, the full expansion of the stent, the radial strength of the stent, the extent of the balloon injury during the stent deployment, the nature of the disease itself (the intensity of the obstruction of the artery), ability to tolerate the compression exerted by the vessel wall, minimum longitudinal contraction by the time of expanding, and the amount of flexibility of the stent, especially for curved vessels to suitably flex in them (Driver 2012; Prendergast et al. 2003; Alexander et al. 2017). The stent material requires to be non-erodible, non-cytotoxic, resorbable, flexible, radio-opaque, biocompatible, compatible with the chemical nature of the drug, and ideally to have sufficient radial strength. Titanium (Ti) and its alloys have been reported as a potential material for the stent backbone with excellent biocompatibility and corrosion resistance as a result of a stable oxide layer on the surface. A new Ti-base alloy was proposed by Saleh et al. as the stent platform with decorated nanostructures on the surface. With comparable mechanical properties to nitinol, Ti-base alloy was investigated as a promising substrate to further replace commonly used stent backbones. So far, there are limited reports on the application of Ti and its alloys for the stent material. However, their coatings showed excellent reduced rate of thrombogenicity and intimal hyperplasia such as Titan stent (Saleh et al. 2017). Another design parameter is the surface coating which must be appropriate for the best adhesion of drugs, be compatible with drug molecules, and be biocompatible (Driver 2012). Generally, materials to be implanted or injected in the body are needed to be both chemically and mechanically stable in the biological environment for long-term use. As a result, when deciding for a potential biomaterial dual approach must be taken: first, studying the biodegradation of the material in the host tissue environment as well as the safety of biodegradation products to impede sensitivity in the local site of prosthesis and second, studying the behavior of the biomaterial during its presence in the body (Guildford et al. 2010). Biodegradable stents are promising candidates for the future vast clinical application. To date, there are two classes of materials for the production of biodegradable stents: metals and polymers. Degradation of the stent, though, is still the most concerning issue due to vessel recoil problems and hypersensitivity (Guildford et al. 2010). The preference of surgeons for using metal-based stents instead of polymeric ones arises from the fact that a metallic platform has not only greater amount of mechanical strength, but also the control over thrombosis rate can be achieved by the association of medications like heparin (Hep) within the stent (Kohn and Zeltinger 2005). In terms of polymer selection, it is important for the coating to maintain the mechanical integrity during DES implantation. Not only the mechanical properties of the coating material, but also comprehensive information on the stent component and their interactions with the host tissue they are implanted in are two other indispensable factors for the safety and efficacy of each stent. Combining the good mechanical properties of the iron metal with biodegradability of polymer employed in metal-polymer composite strategy indicated improved properties of metal-based stents (MBS) in biodegradation rates. The coating of biodegradable aliphatic polyester (PLA) on the ironic metal backbone accelerated the iron corrosion. A complete strength loss of polymer-coated metal-based stents was achieved no more than 6 months in vivo. This degradation rate was much faster than MBS which was attributed to the acidic degradation products of PLA hydrolysis. Animal experiments showed successful tissue regeneration by implanting stents into the animal model (Qi et al. 2017). Extensive in vitro, in vivo and clinical trials are necessary to assure the safety of their implantation for the use in body (Schmidt et al. 2009). First trials for proving the constructed bioconstruction are conducted in vitro by culturing autologous cells onto the so-called scaffold (Generali et al. 2014). By in vitro testing of DES, pharmacological action of drugs can be well predicted (Grabow et al. 2010). Controlling the cell culture under the simulated body condition in a bioreactor is the in vivo test (Generali et al. 2014). In vivo testing of the stent is needed to examine the behavior of a stent in the simulated body condition, but it is not all enough since the results might not be well extensible (translated) for the real application (Grabow et al. 2010). The autologous cells are preferred for this approach to eliminate any probable immunogenic reaction of the body (Generali et al. 2014). So, more long-term follow-up clinical trials are necessary to ensure safety and efficacy of stents.

## Drug delivery mechanism from drug-eluting stents and effective parameters

**Fig. 5:**
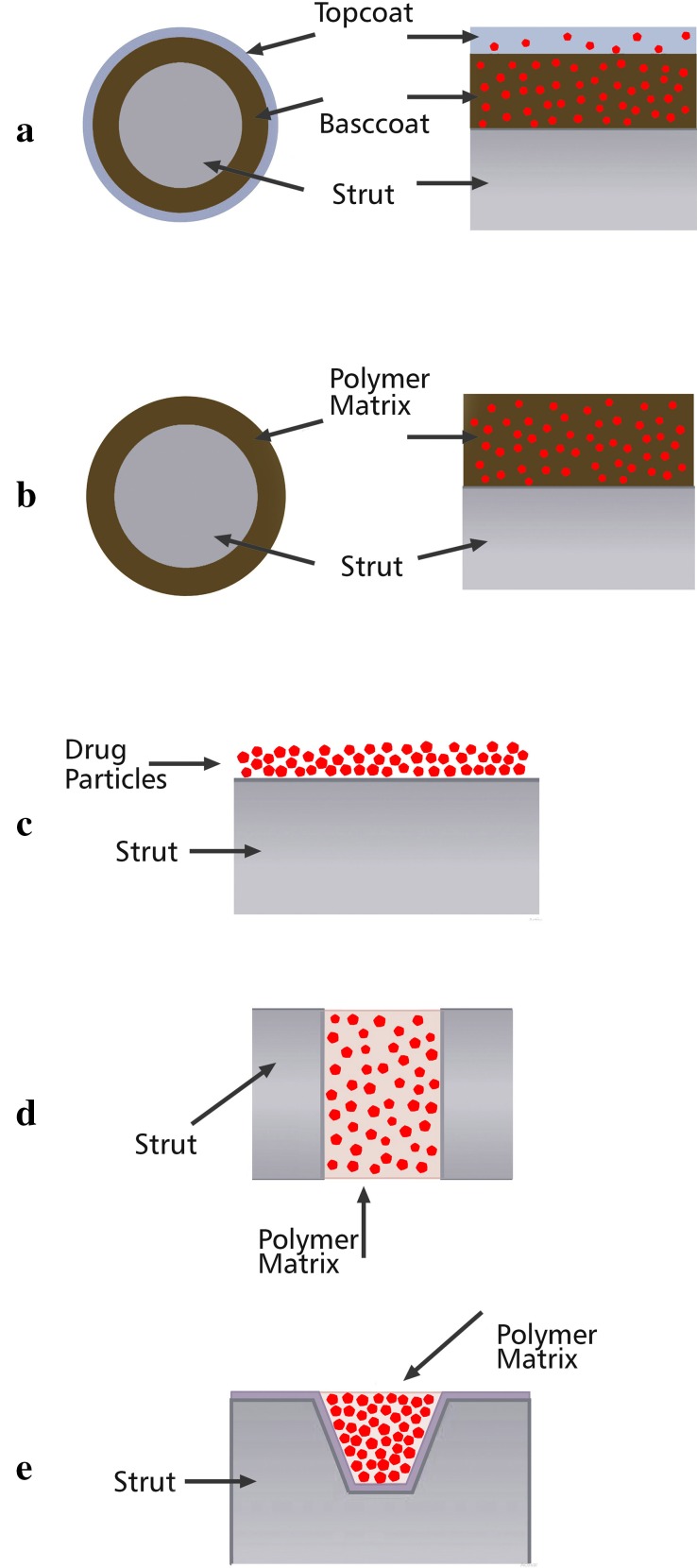
Schematic description of **a** the cross-sectional (left) and side (right) views of a strut of the Cypher stent, **b** the cross-sectional (left) and side (right) views of a strut of the Taxus stent (**a**, **b** are two examples for diffusion-controlled drug release), **c** the side view of a strut of the Achieve stent, **d** the side view of a strut of the Conor stent, **e** the side view of the strut of the Janus CarboStent (**c**, **d**, and **e** are three examples of dissolution/degradation-controlled drug release) (Acharya and Park 2006)

It is imperative to understand the mechanism of drug delivery in order to use the right choice of drug a time-ordered release (Chen et al. 2015). Polymeric systems have been known as efficient drug carriers for two positive reasons including providing a framework for controlled drug release and protecting the drug from degradation before it functions effectively (Martín del Valle et al. 2009). The mechanism of drug release from the polymer substrate can be classified based on the drug–polymer bonding into two major mechanisms of physical and chemical. Physical drug release contains drug release through a permanent polymer layer, dissolution or degradation of the polymer, the permeation pressure, and through an ion exchange process. Chemical drug release is due to the breakage of covalent bonds, which happens as a result of chemical or enzymatic degradation (Hu et al. 2015). The initial drug–polymer system was based on non-biodegradable polymers through which drug diffusion process occurred due to concentration gradient. Later, biodegradable polymers have been used as the major drug-eluting system (Leong and Langer 1988). Polymer swelling, and polymer degradation and erosion are two mechanisms for drug elusion. There are three major mechanisms based on the type of polymer, in which the drug is released including diffusion-controlled system (for permanent polymers), swelling-controlled system (for polymers with the swelling ability), and erosion-controlled system (for biodegradable systems) (Leong and Langer 1988). The main controlled-release devices are classified into reservoirs and matrix systems. In reservoir systems, drug is located in center and is surrounded by a polymeric membrane. Drug agents diffuse through the membrane which is the controlling system. In addition to a membrane form, reservoirs can also be in the form of microcapsules or hollow fibers (Chow et al. 2007). Another form of polymeric system to carry the drug is a matrix device throughout which drug agents are distributed. Matrix devices are more favorable devices to use as drug carrier systems due to prevention of any burst release, and easy fabrication compared to reservoirs. In a diffusion-controlled system it is important for the system to be stable when placing in the biological environment, i.e., not change its size either through swelling or degradation. More importantly, the polymer–drug combination should not induce any change in the polymer structure, and at the same time the drug must be able to diffuse through the polymer pores or macromolecular structure in an effective rate (Langer and Peppas 2003). The schemes of drug release via the surface of five marketed stents including two permanent polymer-coated, one biodegradable polymer-coated, and two polymer-free stents are illustrated in Fig. 5. In swelling-controlled systems, the system is initially dry, but when placed in the body, it absorbs water and swell. This is beneficial to the releasing system regarding disposing any drug release until the device is placed in an appropriate biological environment (Leong and Langer 1988; Nam et al. 2004). Through the swelling process, the polymer free volumes increase and drug diffuses through the swollen network into the site of injury (Martín del Valle et al. 2009). Despite permanent polymeric drug-carrier systems that do not change their chemical structure during drug diffusion, biodegradable polymers degrade within the biological condition after a certain period of time. By degradation, these polymeric drug-eluting systems eliminate the need to be removed from the body after releasing active pharmaceutical agents (Jain 2000). For this superior property over non-degradable polymers, a great deal of research has been conducted on degradable- and erosion-controlled systems (Martín del Valle et al. 2009). There is a difference between degradation which is a chemical process, and erosion which is a physical phenomenon. Erosion can be classified into surface erosion and bulk erosion; the erosion phenomenon is dominantly determined by the chemical structure of the polymer. When the rate of erosion exceeds the rate of water absorption by the bulk of the polymer, the surface erosion occurs. On the other hand, bulk erosion is the drug-controlled mechanism when the rate of water permeation into the bulk is higher than the rate of erosion (Davis 2000). Most biodegradable polymers for delivery system undertake bulk erosion such as polylactide and polyglycolide polymer families (Martín del Valle et al. 2009).

The main advantage with the physical mechanism is that it can be controlled with the designed stenting system. In other words, the stenting system has predetermined kinetics that can be adjusted to a preferred one by changing the efficient parameters. In chemical drug delivery mechanism, grafting drug molecules could result in new chemical bindings which are disadvantages to the system. The chemical mechanism itself is based on the breaking of chemical bonds that bind drug molecules to the system and creates new chemical bonds making the breakage much difficult. In some studies it is far preferred to work with a simple physical mechanism for controlled drug delivery (Chen et al. 2015). The release rate of drugs from the stent surface is directly dependent on the physiochemical properties of the drug (Balakrishnan et al. 2007). Hydrophilicity or hydrophobicity of the drug plays an important role in drug concentration at the site of injury, i.e., hydrophilic drugs were washed away much faster than the hydrophobic drugs. Hydrophobicity will be more favorable since the local concentration in the artery is higher in this case due to their tendency in binding to the structural proteins of the artery wall (Bozsak et al. 2014; Hwang et al. 2001). Not only can the chemical nature of the drug, but also the physical state of the drug to have the crystallized or amorphous structure affect the drug-eluting rate. Crystalline drugs show less rate of dissolution while the amorphous drugs are in higher energetic level, which results in higher drug solubility and faster drug release (Chen et al. 2015). Furthermore, the qualities of drug are considered as an influencing factor for drug release behavior. Qualities of the drug in the physiological medium include the drug diffusion coefficients and drug dissolution constants in the coating of the stent surface, the drug binding rates, and the amount of the transmural convection in the blood vessel wall (McGinty et al. 2014). The coating technique can influence the rate of drug release as an effective parameter (Tan et al. 2012).

## Surface coating techniques

To design a stent, it is vitally important to consider the three-phase biological responses and to apply a time-ordered remedy. The stent surface, therefore, plays a critical role in the success of the implantation. Cardiovascular implants, generally, must meet a biocompatible surface with essential functions of anticoagulation, anti-hyperplasia, anti-inflammation, and pro-endothelialization after the implantation (Li et al. 2017). To elaborate, the surface must have the following requirements: first, to inhibit the inflammatory reaction for impeding the thrombosis formation, to inhibit excessive SMCs proliferation, and to prevent intimal hyperplasia. Second, fast endothelialization from the early time of implantation to promote the formation of endothelial layer on the stent surface within 1 month. Fast endothelialization process is essential to decrease the risk of thrombosis to the least amount. Third, to avoid the adverse material–tissue interface interactions it is necessary for the surface to be biocompatible, especially after complete drug elution (Liu et al. 2014). DES has been initially reported as an effective inhibitor for the migration and proliferation of smooth muscle cells (SMCs) in addition to a healing method to partly treat hyperplasia and inflammation (Kawamoto et al. 2015). However, the loaded drugs on DES were the major cause of late thrombosis due to delayed vascular healing and delayed re-endothelialization (Qureshi and Caplan 2014; Yang et al. 2014). Nanotube-covered stents have been suggested as a feasibility for restoring a functional endothelium and impairing vascular endothelial cells (VSMCs) proliferation (Hemshekhar et al. 2016; Li et al. 2015a; Li et al. 2015b; Zhou et al. 2014). In the presence of nanotube (NT) arrays, it was found that there was an increased proliferation and motility of (VECs) (Hemshekhar et al. 2016; Li et al. 2015a; Li et al. 2015b; Yang et al. 2015). On the other hand, VSMCs showed decreased proliferation and motility in comparison to the control (Hemshekhar et al. 2016; Li et al. 2015a; Li et al. 2015b; Yang et al. 2015). TiO_2_ NTs, as an example, introduced an effective drug-free approach for vascular healing (Hemshekhar et al. 2016; Nio et al. 1993; Yang et al. 2012).Nuhn et al. (2017) provided both in vitro and in vivo data on the implantation of TiO_2_-NT-covered stents (Hemshekhar et al. 2016; Li et al. 2017; Li et al. 2015a). The in vivo results were in agreement with the in vitro study which showed reduced restenosis rates, promoted restoration of a functional endothelium when compared to the control. Restoration of a functional endothelium has important roles in reducing the risk of thrombosis, and alleviating the need for long-term use of anticoagulants and antiplatelet drugs. Their findings reported of the promising results of tissue response after the implantation of the nanoengineered stent. Researchers have demonstrated that ISR, due to intimal hyperplasia, mostly occurred within BMS- while late ST is often seen in DES (Tan et al. 2013). An immunological response due to the implantation of the metal stent is often the leading cause of ISR. On the other hand, late ST is attributed to drug–polymer matrix hypersensitivity and delay in re-endothelialization. New coating techniques and materials for modifying the stent surface are all employed with the intention of reducing the ISR and late ST (Tan et al. 2013). A suitable stent coating is a coating satisfying the physical, biological and regulatory criteria (Luo et al. 2011). To improve the biocompatibility of the stent surface, biomolecule conjugation has been widely applied as an effective method (Jiang et al. 2017). This promising alternative takes great deal of research yet to develop a biomodified surface which possesses cardiovascular associated multi-functions named earlier. In a work done by Li et al., the hyluronic acidpolydopamine (HA/PDA) coating was devised as the surface biomodification. In vivo results have indicated that the excessive high molecular weight HA (HMW-HA) triggered thrombosis, hyperplasia, and inflammation after the imlantation of samples in the pig femoral artery (Wu et al. 2015). One of potential biomolecules to improve surface biocompatibility is hyaluronic acid HA. This natural biopolymer possesses several essential biofunctions which depend greatly on its molecular weight. HMW-HA inhibits platelet aggregation, SMC adhesion, and also macrophage adhesion, and provides vascular cells with anti-coagulation, anti-hyperplasia, and anti-inflammation properties. The problem with HMW-HA is its non-immunogenic property which is unfavorable to implants (Li et al. 2015b; Yu et al. 2016). On the other hand, low-molecular-weight (LMW) HA has the contrary role in comparison to the HMW-HA; it contributes to the inflammation and causes thrombosis (Hemshekhar et al. 2016). In another trial with gradient molecular weight of HA covalently conjugated to a coplymerized film of dopamine and hexamethylendiamine (PDA/HD), the in vivo tests revealed the feasibility of the immobilized HMW-HA (PDA/HD-HMW-HA) for application in cardiovascular implants. PDA/HD-HMW-HA suppressed the adhesion and proliferation of VSMC and the adhesion of macrophages (Jiang et al. 2017).

**Fig. 6:**
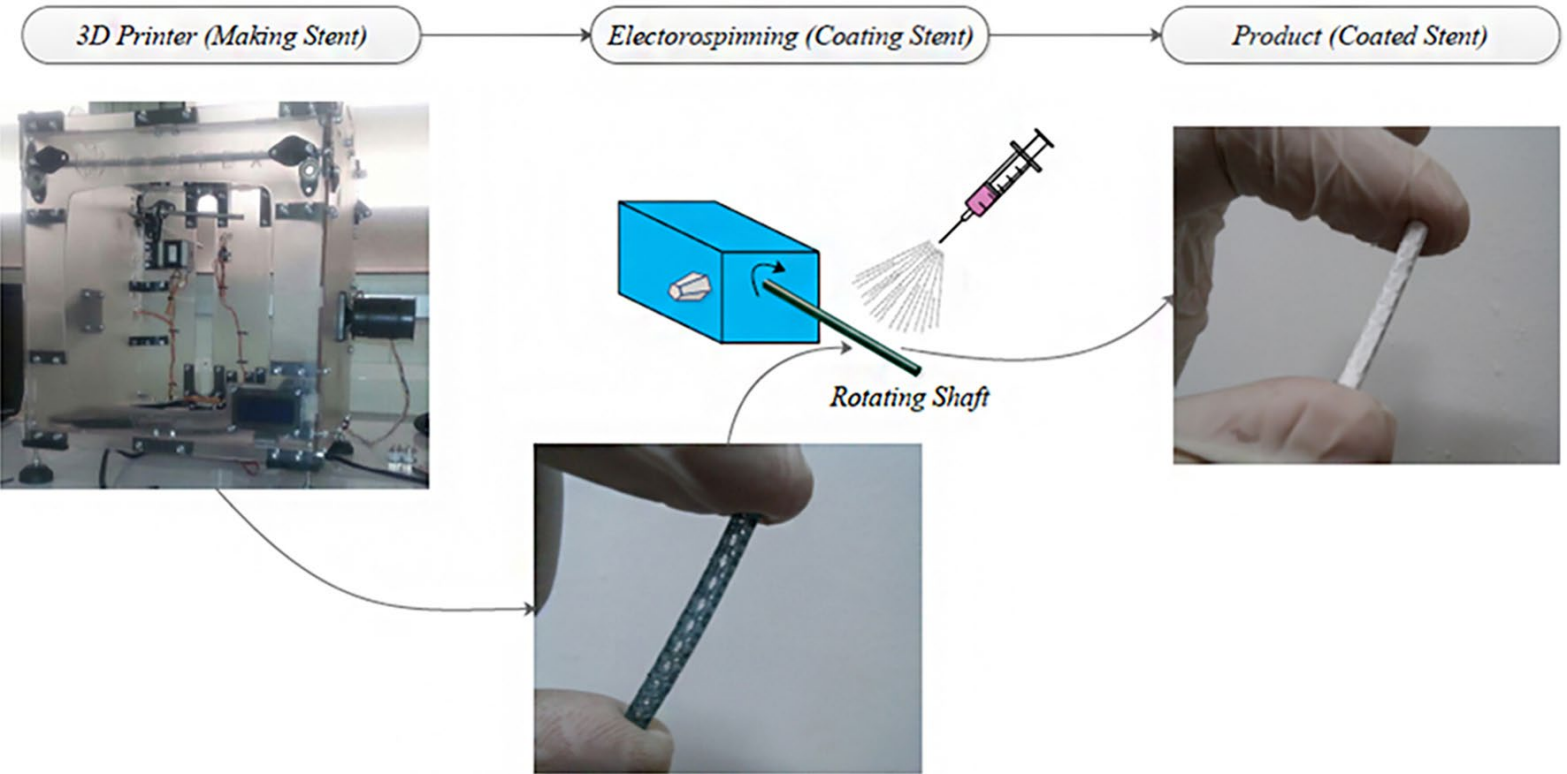
Schematic view of coating procedure

**Fig. 7:**
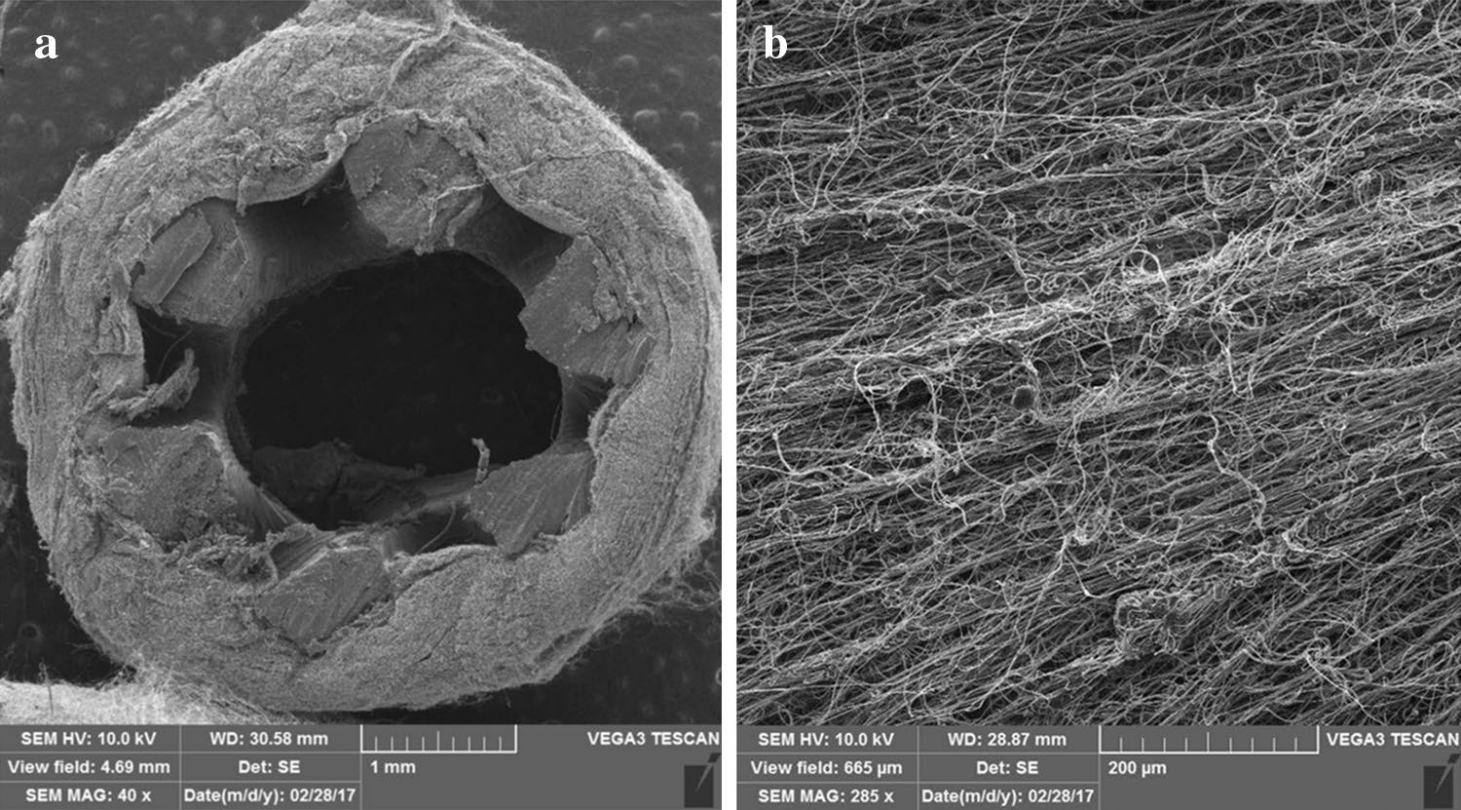
SEM images of the nanofiber-coated PLA stent: (a) cross section and (b) top surface

There are two coating methods for surface modification: physical methods, and chemical methods. The strongly bound (chemical bonds) drug molecules to the surface empowers the DES system with a sustained release rate to the medium while the weakly bound (physical bonds) molecules are burst released (Hu et al. 2015). By chemical methods, chemical bonds between polymer and drug to the stent surface are generated. Generating different groups that chemically bond to the surface usually requires extra treatments to make the surface prepared for further bonding such as anodic oxidation, acid/alkaline treatment or even silanization (Qi et al. 2013). In the physical coating method mostly a liquid solution is applied by dipping, spraying or brushing. Another physical coating technique is the layer-by-layer (LBL) assembly technology in which a nanothin layer of polyanions and polycations can be formed on the charged surface from an aqueous bath (Luo et al. 2011). The NaOH-treated titanium (Ti) substrate was followed by collagen-sulfated chitosan (Col-SCS) multilayers with SCS outermost layer. The evaluation of hemocompatibility of the treated surface was successful and showed superiority when compared to untreated Ti (Li et al. 2009). The well-suited biocompatibility of Ti and its alloys makes this material a good candidate for cardiovascular implants. This is due to the thin layer of oxide film on Ti which is formed immediately after the exposure to the air (Niinomi 2003). The in vitro evaluation of the multilayered Ti showed excellent anticoagulation properties (Li et al. 2009). Another stent surface modification by LBL self-assembly technology was conducted through a study by Wang et al. (Luo et al. 2011) utilizing a biocompatible coating strategy to make the stent compatible with the surrounding tissue; a multilayered drug-eluting stent was prepared through the incorporation of Ch with monoclonal antibody (mAb). mAb antibody can block the platelet glycoprotein (GP) IIb/IIIa receptor and thus prevent platelet aggregation. The LBL self-assembly technology has enhanced the surface properties of the aminolyzed PLLA membrane by mAb/Ch multilayer coating. The mAb release behavior of the system has been improved with this coating technique. Not only the antithrombosis performance of the stent was maintained, but also the healing of the endothelium has been promoted. The in vitro experiments conducted on a PLLA matrix to evaluate haemocompatibility and cytocompatibility of the surface-modified PLLA confirmed biocompatibility of the surface. However, further animal trials are needed to be conducted. For chemical coating method, the silanized Ti surface was covalently bonded to Hep and fibronectin mixture (Hep/Fn). Hep is an anticoagulant agent which is capable of interacting with fibronectin to promote cell attachment and cell proliferation (Adil 1999; Ruoslahti 1988). Thus, simultaneous coimmobilization of Hep and Fn is anticipated to enhance both the anticoagulation and endothelialization of the system. To immobilize covalently active biomolecules on the surface of Ti, the surface was first alkali activated and then silanized to obtain amino reactive groups (Li et al. 2011). Covalently immobilized Hep and Ch mixture on PLA platform was shown as an antiplatelet adhesion which promoted fibroblasts attachment (Zhu et al. 2002). The biomodification of Ti surfaces through the co-immobilization of Hep and Fn gave the system favorable blood compatibility and endothelialization. Immobilized biomolecules are not easily removed by exposure to in vivo condition; however, their bioactivity might be influenced in some cases. In most reported studies, covalently modified surfaces with biomolecules retain the properties of attached biomolecules. The best advantage of immobilization of the surface with these active agents are increasing blood compatibility, or enhancing cell attachment and proliferation which is essential for implanted materials (Li et al. 2011). One suggested solution to resolve this problem is utilizing the simultaneous co-immobilization of anticoagulant and endothelialized biomolecules to improve two properties at the same time and impede any contradictory functions (Uygun et al. 2009). The previous study demonstrated this capability very well. Fucoidan is a sulfated polysaccharide which has been proved as an anti-SMC proliferation far effective than Hep by rat model as an in vitro animal test (Logeart et al. 1997). In addition to this property, there are other pharmacological activities defined for fucoidan including anti-inflammation, anti-coagulant, anti-viral, anti-cancer, and finally anti-peptic ulcer activities. Optimizing coating conditions for fucoidan on a BMS, fucoidan has been suggested an appropriate coating material for the suppression of ISR in comparison to the BMS group (control) (Kim et al. 2015). Carbon-based thin films have been considered an advancement for specific biofunctionalization of stents. Recently, plasma-enhanced chemical vapor deposition method has been suggested to prepare biocompatible carbon-based thin films to prepare the surface for immobilization of bioactive molecules via free-radicals embedded in the coating. Carbon-based films are regarded as capable materials to prevent the adhesion and activation of platelets on metallic substrates (Santos et al. 2015). There are various coating methods available to date, but not all of them are applicable due to following reasons: their limitations in loading drug dose, laboring and difficult programming, long-term drug delivery, and other limitations (Wang et al. 2013a). There is still a strong need for the improvement of coating technology to supply stent manufacturers to optimize drug loading conditions. Despite this fact, recently, there have been novel techniques employed for improving drug release conditions such as utilizing grooves and cavities on the stent struts, constructing nanocarriers like nanopores, nanofibers, nanoparticles, and taking advantage of bioresorbable stent materials with specific drug molecules and utilizing gene NPs to specifically inhibit proliferation of vascular SMCs (Hu et al. 2015; McGinty et al. 2014; Nakazawa et al. 2008). Nanotechnology has been widely utilized to manipulate materials for developing current treatments. They might be applicable in stents and (or) in coating layer of stents. Nanometer drug carriers with excellent biological and physicochemical properties are considered as efficient delivery tools to be used in cardiovascular stents. A drug may be taken up or be covalently bonded to the surface of nanocarriers or even be wrapped into the nanocarriers. Applying nanocarriers, as a stent modified-coating, enhances the localized drug delivery to the injured places. To put it another way, the cell absorption of nanosized drugs outraches the larger size of therapeutic agents. In addition, nanoparticles with much larger surface area in comparison to bulk or microstructures can achieve an effective slow drug release (Hu et al. 2015). In the light of developing nanomodified stents, Alexander et al. (2017) evaluated the ability of peptide amphiphile-based nanomatrix coating for stents under physiological flow conditions in vitro. The results showed the capability of the nanomatrix-coated stent for re-endothelialization, reducing neointimal hyperplasia, reducing restenosis, preventing thrombosis, and alleviating inflammatory response. In an attempt to enhance the efficacy of drug-eluting stents, highly oriented nanotubes were grown vertically on the Ti-based alloy stent platform. This new platform showed potential application for a self-expandable stent. Self-grown nanotubes showed signs of potential as a powerful tool for surface modification to enhance endothelial proliferation, to prevent VSMC proliferation, and also as drug reservoirs (Saleh et al. 2017). Polymer nanofibers have been recently attracted considerable attention and have turned to a hot-spot research focus. The properties of this new coating technique include small pore size, high porosity, large surface area, superior mechanical properties, and the relative easy surface functionality compared to other forms of coating (Morie et al. 2016). High surface/volume ratio increases drug loading capability of the polymer nanofiber as well as cell attachment and drug diffusion (Hu et al. 2015). To attain a heparinized Ti, Hep/poly-l-lysine (Hep/PLL) nanoaperticles were immobilized on a dopamine-coated Ti surface (Liu et al. 2014). The study aimed to reach a coated surface with time-ordered (3-phase) biological function. Through this study Hep/PLL concentration ratio was optimized to control both the Hep immobilized density and release behavior; two deciding factors. The advantages of this functionalized surface were its high anticoagulant activity, selective inhibition of SMCs, and EC proliferation. In vivo animal tests have also demonstrated the predicated time-ordered biofunction to selectively direct an intravascular biological response (Liu et al. 2014). An electrospun surface-coated drug-eluting prototype stent was fabricated by our group. The electrospun composite nanofiber made from PLLA, Ch, and PTX surface covered the previously in-lab fabricated 3D-PLLA stent by means of a single nozzle electrospinning approach. The scheme of our experimental procedure is shown in Fig. 6. To achieve the optimum composite fiber drug concentration and Ch concentration were found as effective parameters. The 40 and 60% PTX concentrations were found to show excellent biocompatibility for the system. SEM images (Fig. 7) have also confirmed the effectiveness of PTX in reduction of cell proliferation on the composite nanofiber-coated PLLA. Structural results have confirmed the proper encapsulation of PTX into the polymeric matrix of PLLA with Ch in combination. There has been no drug–polymer chemical reaction to decrease the PTX biological function. Drug release pattern showed a burst release (10–15% of total mass of the drug) in the first day followed by a sustained release behavior (Khashi et al. 2018).

In another work, a polyhedral oligomeric silsesquioxane poly(carbonate-urea) urethane (POSS-PCU) nanocomposite polymer was successfully coated on the BMS surface with anti-CD34 antibodies covalently attached to the POSS-PCU. Covalent attachment of anti-CD34 antibodies on the surface of POSS-PCU enhanced capture of circulating endothelial progenitor cells. This ability did not affect the in vitro biocompatibility and haemocompatibility of the coated-metal stent (Tan et al. 2013). The idea of utilizing POSS-PCU as the nanocomposite polymer coating on a BMS lies in three first-in-man implants (Ahmed 2011; Chaloupka et al. 2011; Jungebluth et al. 2011) for biomedical studies reported earlier. The success of the previous human implants implied the unique and suitable properties of this nanocomposite as an ideal candidate for coating medical devices by Seifalian’s research group. Recently, hormone therapy has been considered as one of alternative treatments for cardiovascular diseases (Schierbeck et al. 2012). The general idea lies on the functional property of hormones for regulation of reactive oxygen species (ROS). It is found that ROS can induce the pro-coagulant process and the increase of the ROS level can induce SMS proliferation which causes thrombosis. β-Estradiol is a female hormone which has been utilized as an anti-inflammatory and anti-thrombosis drug in stents, recently (Oh and Lee 2013). The electrospun nanofiber surface-coated stent containing this hormone showed a high endothelial proliferation rate and efficiently regulated the ROS. The electrospun solution contained the mixtures of hexafluoro-2-propanol, PLGA, and PLA as a nanofiber base and nanoparticles containing β-Estradiol as a drug and eudragit S-100 as the polymer to carry the drug. Those nanofibers incorporated with β-Estradiol promoted much higher endothelial proliferation rate, about threefolds greater than the nanofibers without β-Estradiol (control). The results of this study predicted this stent as a potential cardiovascular implant for the blocked vessels in the future (Oh and Lee 2013). The first human trial with estrogen-eluting stent (hormone-eluting stent) showed the optimistic result of lower rate of restenosis (Abizaid et al. 2004). On the other hand, two clinical trials did not obtain the same success as the human trial. In these randomized trials β- Estradiol was used as the drug and could not exert enough anti-inflammatory activity (Abizaid et al. 2007; Airoldi et al. 2005). The failure of the hormone as an effective pharmaceutical agent in previous trials was attributed to the following reasons: first, low dose of β-Estradiol inhibited the efficiency of the hormone as a drug. Second, the coating process has cast a shadow on the biological effect of β- Estradiol (Ryu et al. 2009).

## More new stent systems

### Shape-memory stents

**Fig. 8:**
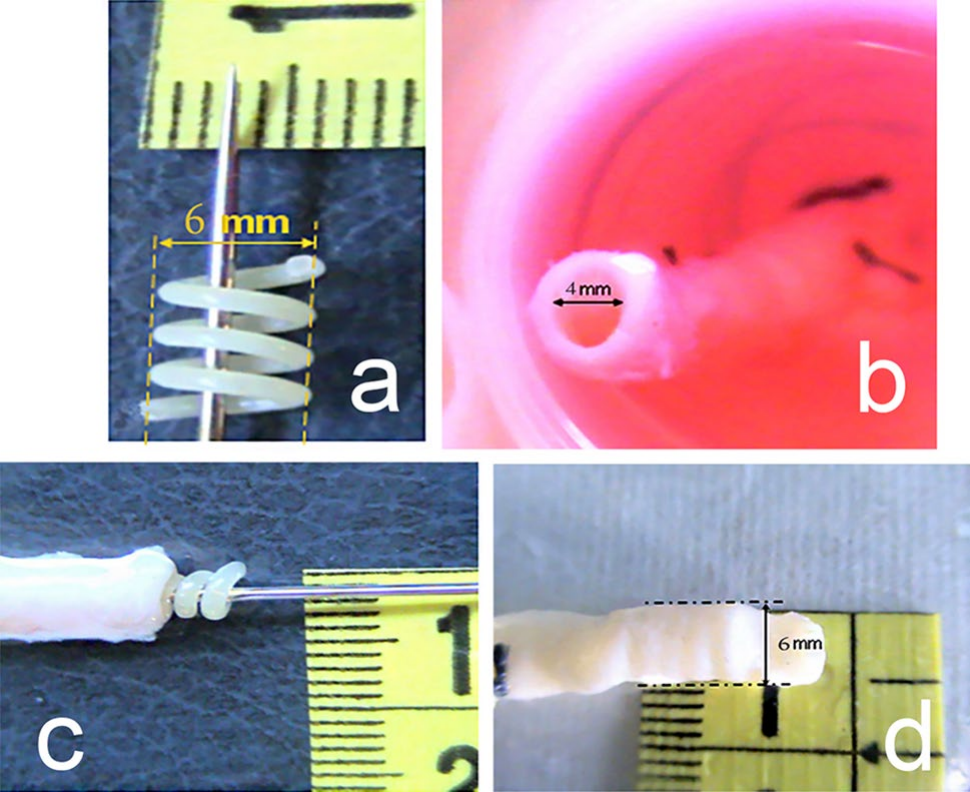
Performance of the proposed screw stent made of PU/PCL (70/30) blend **a** in a segment of femoral vein removed from the leg of a patient, **b** stent in temporary shape (a screw with very small diameter) over a delivery instrument, **c** recovery step of the SMP stent delivered into the segment of 4 mm ID human femoral vein containing body temperature water at 37 °C (**d**). The filament diameter of the stent is 800 µm

**Fig. 9:**
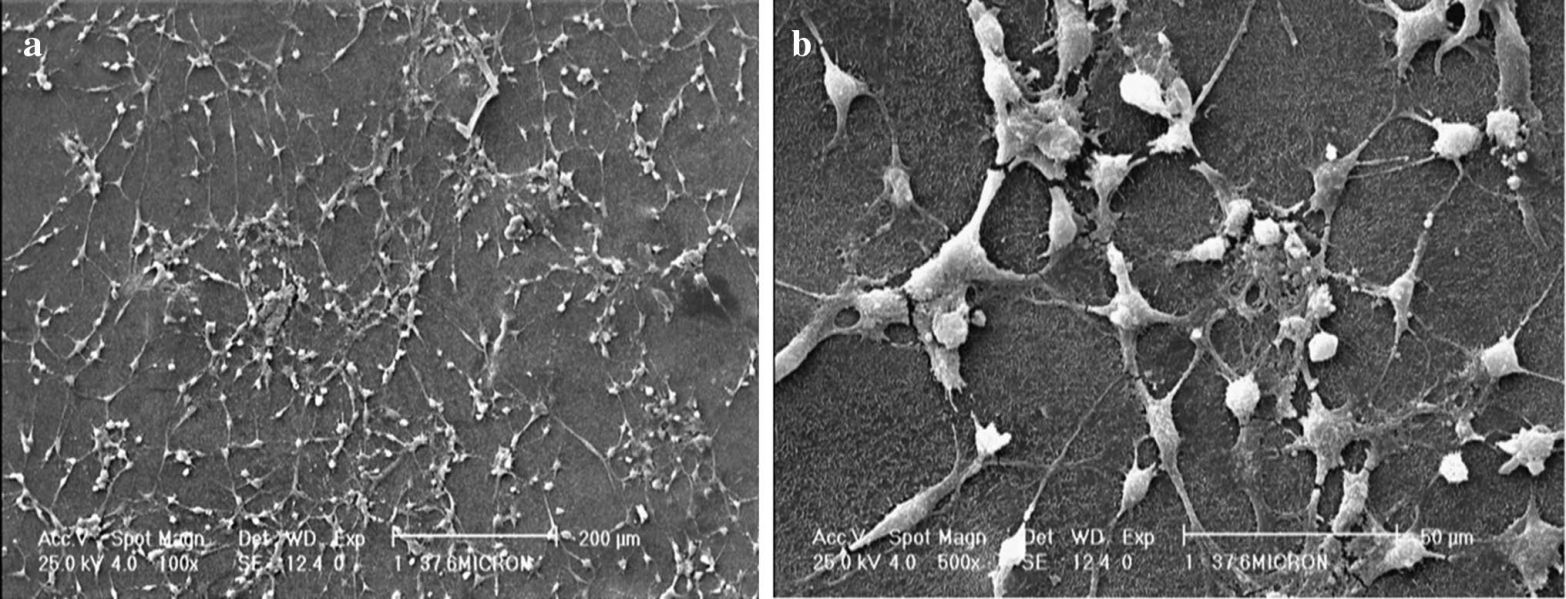
SEM images of NIH-3T3 cells proliferated on PU/PCL(70/30) at day 7 **a** initial magnification *100 **b** initial magnification *500

**Fig. 10:**
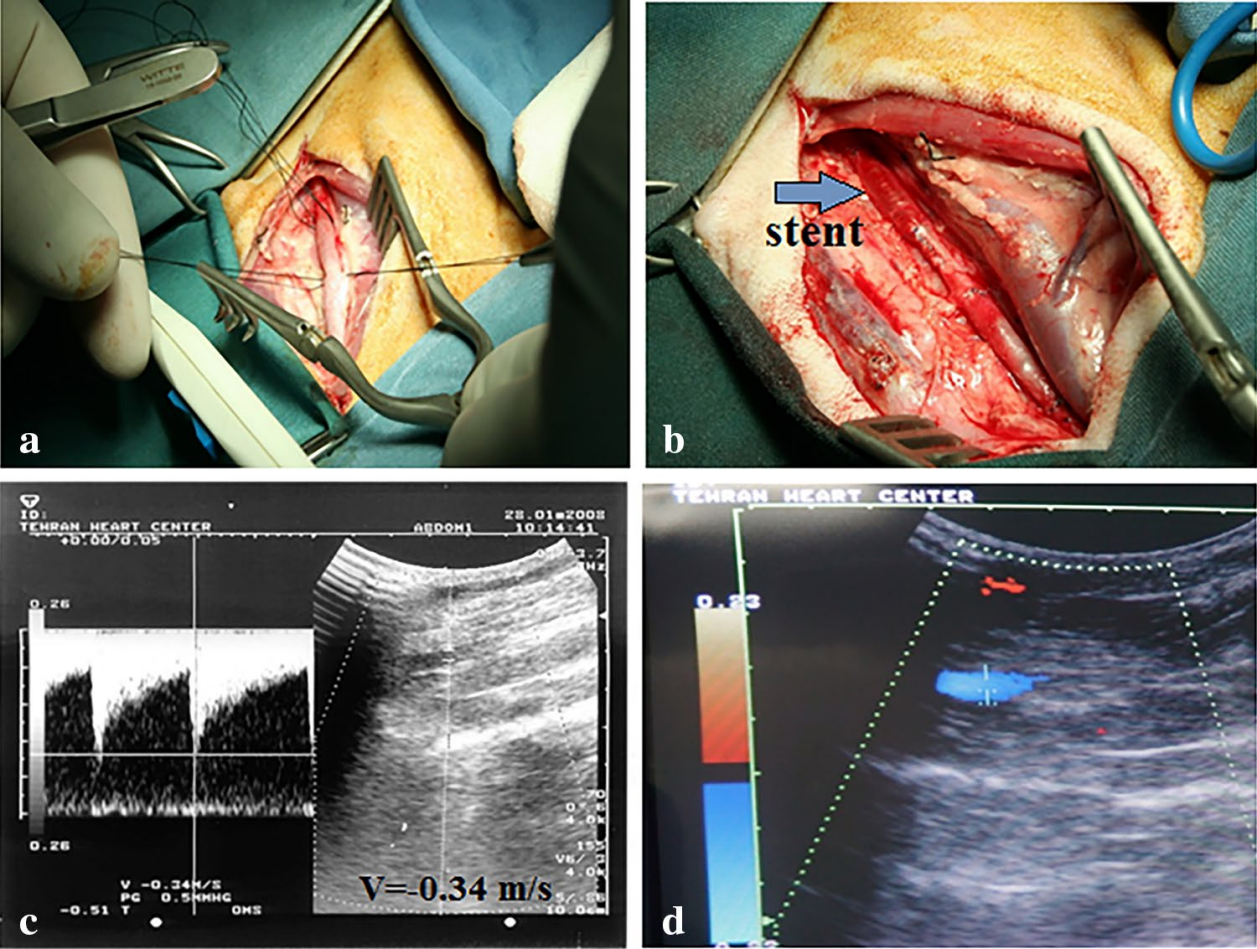
Carotid artery of the sheep model: **a** before and **b** after the shape memory stent deployment; **c** transverse gray-scale and **d** color Doppler sonograms of the carotid artery at 4 months after stent placement

Another type of stent which might play a part of future trend as the basic technique is shape-memory stent. It has the ability of self-expansion in the range of body temperature in an ideal situation (Ajili et al. 2009). Shape-memory polymers (SMPs) are stimulus-responsive materials that change their shape in response to an external stimulus. They contain a 2-phase shape transition: in the first phase, the polymer is fixed in a temporary shape. In the second phase, the polymer is stimulated by an external stimulus to recover its permanent shape. One of the stimulus which makes a category of SMP is heat, and the type of polymer, which its phase transition is induced by external heat, is called the heat-induced shape-memory polymer (Yang et al. 2013). Most of SMPs are thermally vulnerable which is known as thermo-responsive SMP. In other words, there is a critical temperature in which their temporary deformation is eliminated and permanent shape can be recovered (Ping et al. 2005). The temperature in which this type of polymer has the transition to reach the permanent shape is the transition temperature. The most favorable ones to be applied in biomedical applications are those with the transition temperature around the human body temperature (Yang et al. 2013). Bulky devices with this ability could be a potential candidate to be implanted in the body with a compressed temporary shape which then expand to their permanent shape (Lendlein and Langer 2002). There are reports for evaluating the performance of non-biodegradable SMPs like the work of Yakacki et al. (2007), in which they made use of *tert*-butyl acrylate and poly(ethylene glycol) dimethacrylate. Although the stent was activated at body temperature, however, the stent was made of non-biodegradable materials. The driving force for SMP to be used as a substitute for metallic framework of stents is mainly the long-term complications with metallic stents within the vessel. Biodegradable SMPs might be an ideal alternative choice for use as the future generation of cardiovascular stents (Yang et al. 2013). The first human-implantad stent was Igaki-Tamai which was made from PLLA (Tamai et al. 2000). The self-expandable stent changed from its temporary shape into the permanent only if it was heated above the switching temperature of 70 °C. In Igaki-Tamai, the stent delivery balloon inflation was performed with a heated dye at 80 °C. Another self-expandable stent made use of the same heating method of the Igaki-Tamai stent was PCL stent reported by Peng et al. (1996). In addition to this type of heating-derived expansion, they also suggested microwave and electrical heating in the balloon for stent self-expansion. However, the mandatory heating could injure cells during the stent deployment. It is most recommended to implant the self-expanded stents near the body temperature (Venkatraman et al. 2006). This would be feasible with a 2-phase polymer transition in shape: first, the pre-transition in which the polymer has a temporary shape; and second, the post-transition in which the polymer recovers its permanent shape. To the best of our knowledge, our group was the first to report the use of polyurethane/PCL (PU/PCL) blend as a proposed material for a SMP-based stent (Ajili et al. 2009). To achieve a body-range self-expandable polymer stent, we made use of the elastic memory ability of this blend. Shape recovery of the blend takes place at the melting temperature of PCL crystals which are formed during the deformation and fixation. Effective parameters to adapt the shape recovery of the blend in the range of body temperature are composition of the blend and crystallization conditions. The performance of this stent was evaluated in a segment of human femoral vein at 37 °C as shown in Fig. 8. The blend showed excellent tissue compatibility by examining the adhesion and proliferation of bone marrow mesenchymal stem cells (Fig. 9). Our results suggested that PU/PCL blend with the weight ratio of 70/30 could be a potential candidate as a stent material for future. Self-expandability in the range of the body temperature is of critically challenging issue to the SMP-based stents. Our group has successfully surpassed this challenge. In this approach (Ajili et al. 2009) we have applied crystallinity-induced shape-memory effect to incorporate elastic memory in the stent. In addition, an in vivo experiment was done on the carotid artery of a sheep model to determine the feasibility placement of the prepared shape memory stent, its mechanical strength and radial stiffness for opening the vessel lumen. Briefly after the prep and drape of the neck, a 10-cm incision was made on the left side of the neck and carotid artery was exposed. Two clamps were put at the proximal and distal of the carotid artery and an incision made to place the stent in the carotid artery. Then the carotid was sutured and clamps removed to let the blood flow. Muscles and skin were closed and the animal has recovered. Sonography was used to evaluate the carotid artery and visualization of the stent diameter after its placement. The sheep model^2^ which was followed for more than 4 months demonstrated an enough radial force to dilate and open the lumen and good hemocompatibility according to sonographic criteria and normal color sonogram images on follow-up (Fig. 10) (Ajili 2008).

In a study by Yang et al. (2013), they developed a thermo-induced, biodegradable shape-memory stent. The cross-linking PEG-PCL-based stent was able to transient from a temporary linear form to a permanent spiral shape with the transition temperature close to the body temperature. The combination of drugs including mitomycin C as an anti-proliferative drug, and curcumin as an anticoagulant agent in collaboration with the shape memory polymer could successfully serve as an effective device to treat the coronary artery disease. This biodegradable stent maintained its integrity with a 47% weight loss after 2 months keeping in the simulated body condition proving not being collapsed into parts during bulk degradation. In another report by Chen et al. (2007), a biodegradable stent with the shape-memory property was developed. This stent was made of a blended chitosan (Ch) film cross-linked with an epoxy compound. To evaluate the efficacy of the prepared stent a commercially available metallic stent was used as a control. The results indicated the supremacy of polymeric stent in comparison to the metallic stent in respect to the elastic deformation. The metallic stent could tolerate elastic deformations of 10% before deformation; however, the polymeric stent could tolerate deformations up to 30% and after that it recovered the permanent configuration. The shape transition from a crimped (temporary shape) to a fully expanded state (permanent shape) took about 15 s which was advantageous to the system to avoid any migration from the location. Venkatraman et al. (2006) reported of a self-expandable stent with the feature of expanding at the body temperature. This bilayered biodegradable stent was composed of PLLA and PDLGA. In this study (Chen et al. 2007), the research team made use of glycerol and PEG (or PEO) to blend with Ch in order to improve the mechanical strength of Ch. More importantly, to achieve the shape-memory property, blended Ch was cross-linked with ethylene glycol diglycidyl ether as an epoxy compound in an aqueous environment. The bright side of this study is the reversible shape-switching process through hydration or water desorption. The fundamental drawback of this study is the long duration of full expansion of the stent after deployment. The minimum time of full expansion of the stent was 8 min which increased the possibility of stent migration during deployment. To avoid this problem, the stent expansion after the insertion should be fast.

### Polymer-free drug-eluting stents

**Fig. 11:**
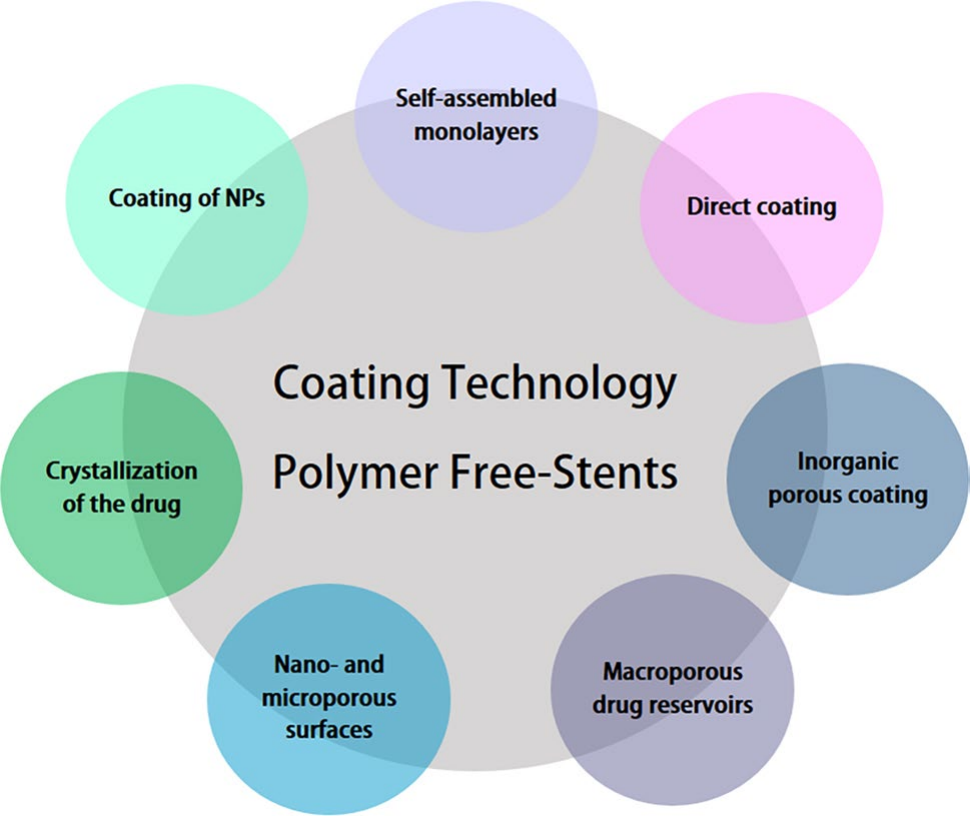
Schematic representation of the techniques used to manufacture polymer-free DES (Chen et al. 2015)

Despite of favorable effects with DESs, clinical studies have proven the inflammatory triggering effects of toxic ions generated from the degradation of polymeric coatings or degradable metal or metal alloys as surface coating (Navarese et al. 2014). An option to completely make rid of polymers as the drug-carrier is to develop a polymer-free stent. This alternative should be able to preserve functions of polymeric DESs including carrying drug molecules, binding the drug to the stent, controlling the drug release rate at a suitable rate (Acharya and Park 2006). More importantly, carrier-free stents need to be biocompatible to be adapted to the tissue surrounding. In comparison to polymeric coating as the drug-loading platform, polymer-free stents are expected to have a faster drug elusion rate which might have adverse therapeutic effect. However, the efficacy and safety of the latter stent is comparable to the first-generation DES. Although polymer-free stents have been performing well in preclinical and clinical trials, these stents do not outperform second-generation DES, yet. There are seven coating technologies for polymer-free stents including direct coating, crystallization of the drug, nano- and microporous surfaces, inorganic porous coating, macroporous drug reservoirs, coating of nanoparticles, and self-assembled monolayers (Chen et al.^2015^). Thiruppathi and Mani (2014) named a same list of 7 coating technologies in a different manner including molecular coatings (Gallo and Mani 2013), self-assembled monolayers (Mani et al. 2011), microrough surfaces (Lancaster et al. 2012), porous surfaces (Tsujino et al. 2007), textured surfaces (Wessely et al. 2005), and reservoirs (Morice et al. 2006). A schematic representation of these coating techniques is shown in Fig. 11.

Thiruppathi and Mani (2014) made use of vitamin-C (l-ascorbic acid, l-AA) as an anti-proliferative drug to promote endothelial cells’ growth faster than the conventional anti-proliferative drugs. In their study, a CoCr alloy surface was used as the stent platform and an auxiliary platform for loading l-AA, namely a polymer-free phosphoric acid platform and polymer-based-PLGA platform. The potential ability of vitamin-C (l-AA) to treat as a therapeutic drug over anti-proliferative drugs such as sirolimus for encouraging endothelial cells growth has been demonstrated in the previous study (Kakade and Mani 2013). Despite successful l-AA coating onto CoCr alloy surfaces, results of drug release from two distinct platforms of polymer-free and polymer-based platform were not the same. l-AA was burst released from the polymer-free CoCr alloy surface for 1 h. However, l-AA was sustained-released from PLGA coated CoCr alloy surface for 24 h (Thiruppathi and Mani 2014). Drug-eluting process is confirmed to be an inevitable mechanism for stents today. Making use of nanotechnology, mesoporous silica nanoparticles (MSNs) have been regarded as excellent drug-carrier for polymer-free stents for the following properties: first, tunable pore size; second, high specific surface area; third, large pore volume; and finally, their biocompatibility to the tissue (Popat et al. 2011; Vivero-Escoto et al. 2010; Zhang et al. 2008). In a study by Wang et al. (2013b) a novel polymer-free DES stent was constructed making use of magnetic MMSNs and carbon nanotubes. The nano-structured coating on the stent platform has resulted in a new polymer-free stent that exhibits excellent mechanical flexibility, blood compatibility with a satisfactory drug releasing profile. Rapid endothelialization, which is important as the inhibitor of late ST, has been achieved through the coated composite layer on the stent platform. In their previous studies Liu et al. 2012; Rosenholm et al. 2011; Zhang et al. 2012 employed core–shell MMSNs as the drug-carrier system and were the first to report on the use of MMSNs as a coating layer on cardiovascular stents experimented in vivo. The disappointing result, though, was within low flexibility and mechanical failure of the coating during stent expansion. A promising report was within a study (Alviar et al. 2012; Costa et al. 2009) in which a nanothin-microporous hydroxyapatite surface-coated stainless steel stent was used as a polymer-free DES for releasing sirolimus. The low-dose drug-loaded polymer-free stent has shown satisfying results for 1 year clinical trials. Recently, there has been a report on drug delivery and capability of DES-based crystallized coating. The polysaccharide top coating was applied onto a Rapamycin (RM) crystalline layer. The top coat was a protective layer for the crystalline layer to suppress delamination during the stent crimping and expansion. The crystalline top coating enhanced the quality of the carrier-free stenting system regarding the physical, mechanical, and chemical stability of the stent (Farah 2018). Although all these efforts had advantages to the development of polymer-free stents, designing new coating materials and structures is still needed. Nanotechnology could be a beneficial technology for promising results in this field. The modification of these stents brought both its own advantages and disadvantages the same as all previous stent technologies. They have improved the duration of drug release from the stent although they have failed in mechanical integrity required for stent backbone in most cases (Hsiao et al. 2013). Polymer-free stents also indicated a capability for long-term safety in comparison to traditional coated DES (Farah 2018). There is still no guaranteed success, though, and more research studies need to be developed.

## Conclusion and future perspective

Despite a revolutionary role of endovascular stents in terms of clinical outcomes in the interventional cardiology, complications associated with stent implantation have still remained a major problem. To overcome the limitations, there have been many reports as the treatment option of choice for stent design. These reported treatments can be categorized into a main list which includes the following attempts: Surface modification with improved polymer coating materials, nanocomposite materials, and tissue engineering-contributed strategy to improve surface properties. There are also affairs to improve the stent backbone like brand new covered stents, polymer-free stents, brand new bioresorbable and biodegradable materials.

In marketed DES, a therapeutic coating agent is applied together with a polymer carrier which is responsible for holding the drug mechanically, preserving chemical stability of the drug, and regulating the drug release profile. Safety and clinical issues have become the main obstacle to get the FDA approval. So far, polymer properties have not satisfied all the required properties, and there is far development to achieve a polymer structure with all advanced properties at once. Coating durability, physical properties adapted to the vascular tissue medium, compatibility with the drug nature, biocompatibility, controlled and sustained drug release are key requirements for polymers to be applied in stents. In practice, it is almost impossible to have all desired properties come in one polymer. So, the mixture of polymers is almost used to bypass limitations. New technologies are under development to take advantage of biodegradable polymer-based stents. Polymeric nano-formulations have been considered as an alternative on the table to find their practical way into the clinical practice in the future. Currently, some research has led to the evaluation of the capability of carrier-free DES as one of the future possibilities. Polymer-free drug-eluting stent has been first designed to make up for increased risk of thrombosis and inflammation ascribed to polymers. Advanced design of polymer-free stents leverage porous surfaces and reservoirs to inscribe substantial amounts of anti-inflammatory and immunosuppressive drugs. Preclinical and clinical trials have reported the comparable performance of polymer-free stents with stents coated with polymer (Chen et al. 2015). The carrier-free stent technology has indicated the prospective long-term benefit and increased efficacy over traditional polymeric DES (Farah 2018). The BRS technology is still in its infancy and further development needs to be conducted to optimize the technology to find a way to a safe market. BRS of the future, without doubt, is more likely to impact many aspects of the recent interventional cardiology. Something that is clear about BRS is that the future BRS generation needs to have a safe implantation with higher mechanical stability. To our knowledge, papers until recently have reported no available evidence on the development of a marketed resorbable stent with no thrombosis incidence. Given the current reports, there seems to be no satisfying clinical reasons to choose one of the novel stent designs over the current in-use stents. Bioresorbable devices need a clear clinical benefit in comparison to DES but published data have not yet confirmed any outperformance of what was expected from the modern DES. More importantly, many of the already suggested and worked-out designs have lack of sufficient clinical results and are still under in vivo experiments. We can generally frame our prediction of the next revolutionary stent on the therapeutic potential of nanobiomaterial-based platforms serving as a drug-releasing treating system. Biomolecule-decorated polymeric surfaces in association with nano-devised techniques would be the most common strategy for the future applications.

## Footnotes

1 ABSORB is a bioabsorbable everolimus-eluting coronary stent system, Abbott Vascular Co., Santa Clara, CA, USA.

2 All the experiments were performed according to the policies of the Institutional Animal Care and Use Committee at Tehran University of Medical Sciences in accordance with the NIH Guide for the care and use of laboratory animals (NIH publications, 1996).
